# Low-temperature (< 200 °C) degradation of electronic nicotine delivery system liquids generates toxic aldehydes

**DOI:** 10.1038/s41598-021-87044-x

**Published:** 2021-04-08

**Authors:** Nicholas R. Jaegers, Wenda Hu, Thomas J. Weber, Jian Zhi Hu

**Affiliations:** Pacific Northwest National Laboratory, Richland, WA 99354 USA

**Keywords:** Risk factors, Chemistry, Organic chemistry

## Abstract

Electronic cigarette usage has spiked in popularity over recent years. The enhanced prevalence has consequently resulted in new health concerns associated with the use of these devices. Degradation of the liquids used in vaping have been identified as a concern due to the presence of toxic compounds such as aldehydes in the aerosols. Typically, such thermochemical conversions are reported to occur between 300 and 400 °C. Herein, the low-temperature thermal degradation of propylene glycol and glycerol constituents of e-cigarette vapors are explored for the first time by natural abundance ^13^C NMR and ^1^H NMR, enabling in situ detection of intact molecules from decomposition. The results demonstrate that the degradation of electronic nicotine delivery system (ENDS) liquids is strongly reliant upon the oxygen availability, both in the presence and absence of a material surface. When oxygen is available, propylene glycol and glycerol readily decompose at temperatures between 133 and 175 °C over an extended time period. Among the generated chemical species, formic and acrylic acids are observed which can negatively affect the kidneys and lungs of those who inhale the toxin during ENDS vapor inhalation. Further, the formation of hemi- and formal acetals is noted from both glycerol and propylene glycol, signifying the generation of both formaldehyde and acetaldehyde, highly toxic compounds, which, as a biocide, can lead to numerous health ailments. The results also reveal a retardation in decomposition rate when material surfaces are prevalent with no directly observed unique surface spectator or intermediate species as well as potentially slower conversions in mixtures of the two components. The generation of toxic species in ENDS liquids at low temperatures highlights the dangers of low-temperature ENDS use.

## Introduction

Since electronic nicotine delivery systems (ENDS, vape, or e-cigs) entered the consumer market in the early 2000s, they have become an increasingly popular alternative to tobacco-based products, often marketed as a safer and healthier alternative which assists with cessation^1^. Such products rely on the heating and subsequent vaporization of liquids over an atomizer (typically a resistively heated Clapton coil composed of kanthal, stainless steel, nichrome, nickel, or titanium). The aerosols are inhaled into the lungs where the chemicals may directly interact with pulmonary cells to modulate physiological responses. ENDS liquids are typically comprised of propylene glycol, glycerol, nicotine (1–3%), and flavoring agents (< 1%). The two main ingredients, propylene glycol and glycerol, are typically regarded as safe for human use; however, the heating process exposes these liquids to elevated temperatures to stimulate the vaporization process and may also result in the thermal oxidation of the ENDS liquid components. Indeed, there is growing evidence that suggests such a process may be playing a role in the negative health effects that have arisen from ENDS use in recent years. Namely, the combustion of the liquid components to form a variety of potential toxins such as formaldehyde, acetaldehyde, propanol, carbon monoxide, acrolein, o-methyl benzaldehyde, nitrosamines, limonene, radical species, and other chemicals coupled with inhalation of volatilized metal particles and metal carbonyl complexes^2–5^. Exposure to such compounds is associated with adverse health effects, such as the decline in cardiovascular health in smokers and potentially even to individuals exposed secondhand^6,7^. Numerous factors impact the extent to which these compounds form, such as the liquid composition, power output of the resistively-heated coil, puff duration, and the coil material and configuration^8–11^.

A further complexation is the environment under which ENDS liquids are heated, i.e. the liquid composition, temperature, and carrier gas. Comparison of traditional cigarettes to ENDS has shown that carbon monoxide is still present even in the absence of combustion^3,12–14^. Such product formation has been attributed to pyrolysis-like conditions in the ENDS atomizer. It has been shown that anaerobic conditions result in reduced degradation relative to air-drawn ENDS. Under such anaerobic conditions, propionaldehyde and acetone are key products of propylene glycol pyrolysis at temperatures around 413 °C, likely from dehydration. In contrast, a wider array of compounds arises from oxidative conditions due to partial or complete oxidation^15^.

Such processes can occur spontaneously at elevated temperatures, but the use of a catalytic surface often enables such transformations at lower temperatures than that of auto-ignition. It has been shown that the ENDS coil itself may serve as such a catalytic surface for these oxidation and/or degradation reactions. Both kanthal and stainless-steel coils, for example, have been shown to stimulate the oxidation of propylene glycol at temperatures as low as 256 °C, where reactivity on nichrome was favored at temperatures between 360 and 560 °C^15^. At these temperatures, oxidation of ENDS liquids to toxic products is favorable. Much of the high-concentration toxicant emission have been attributed to dry puffing, a mode of operation in which the atomizer filament is ill-saturated with liquid and reaches high temperatures. Atomizer coils operate under a wide range of temperatures from 110 to 1008 °C, depending on the duration of the inhalation, coil resistivity, power output, ENDS liquid composition, and coil wetness^16^. Lower temperatures (110–185 °C) are typical of conditions when the coil is in direct contact with liquid and typically regarded as inert with respect to ENDS liquid degradation^15,16^. Under saturated wick conditions, temperatures are typically observed between 145 °C and 334 °C. Dry puff conditions have been an area of great concern since temperatures can reach as high as 1008 °C and stimulate the formation of a wide array of high abundance toxicants. Indeed, it was shown by inline infrared spectroscopy measurements that higher temperatures lead to a much larger degree of CO, CO_2_, methane, ethylene, and acetylene formation^2^. The focus on the thermal decomposition of ENDS liquids have examined relatively higher temperature ranges since this produces higher concentrations of toxins in aerosols. The oxidation of glycerol has been studied via infrared spectroscopy on gold nanoparticles at low temperatures for the selective formation of tartronic acid^17^, which was not observed herein. In the present work we show that degradation of ENDS liquid components can be observed at temperatures below 200 °C that are more consistent with direct liquid contact vaporization. At these relatively low temperatures, reactivity is inhibited and may fall below the detection limit of previously selected analytical techniques given the short duration of the thermal treatment. However, chronic exposure to low levels of toxins may still occur with a potential to negatively impact users’ health.

**Figure 1:**
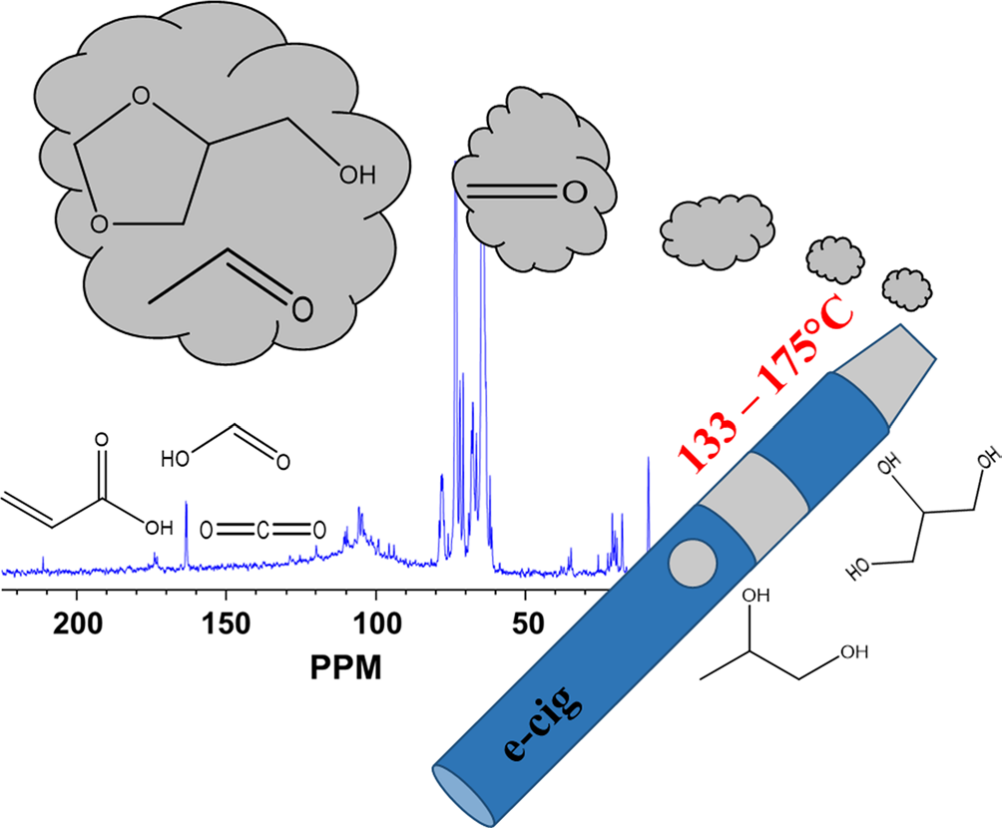
Representation of monitoring the low-temperature thermal degradation of ENDS liquids via NMR.

**Figure 2:**
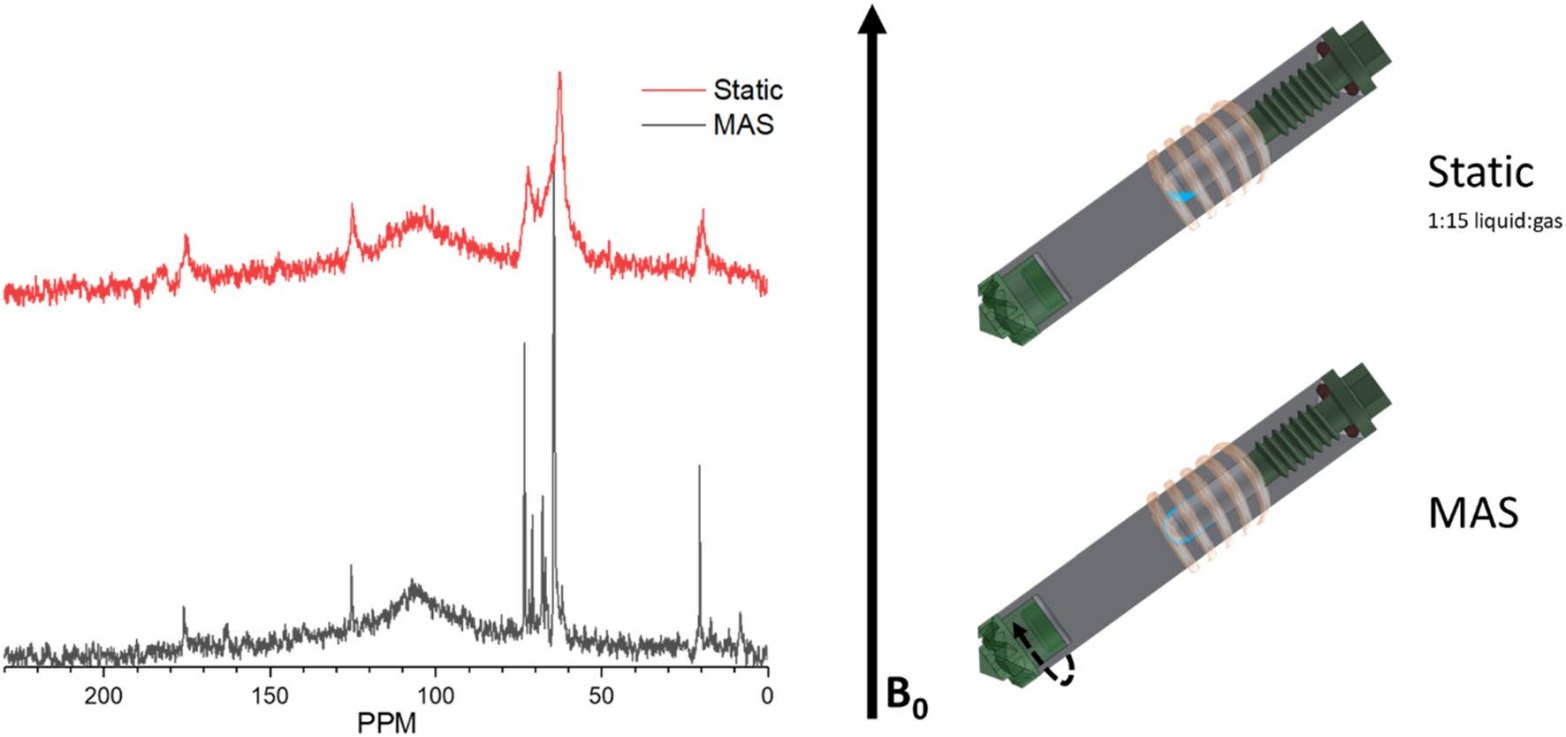
Demonstrative ^13^C NMR of 20 μl of glycerol and 75 psig O_2_ reacted over 50.1 mg dehydrated ZrO_2_ at 175 °C with and without magic angle spinning (left) and cartoos of the rotor and approximate contents inside the coil and magneti field (right). The static spectrum was acquired with 12,000 scans and the spinning was collected with 32,000 scans. The NMR rotor representation was created using Solidworks (www.solidworks.com).

Model compound investigations have primarily used propylene glycol degradation as the primary focus for much of the available literature^15^, though some studies have included mixtures of propylene glycol and glycerin^14^. Herein, we investigate the isolated components (propylene glycol and glycerol) and mixtures of both constituents under tight control by in situ ^1^H and ^13^C NMR, without introducing enhanced ambiguity from nicotine or flavoring agents in this work. Magic-angle spinning (MAS) nuclear magnetic resonance (NMR) is a non-destructive analytical tool which can monitor the transition of ENDS liquids to toxic compounds at a specified composition, pressure, and temperature. A major strength of this approach lies in the fact that NMR can often be used to analyze the intactions of chemical species at relevant temperatures without the need to heat or ionize the species for analysis and potentially cause degradation or fragmentation (as might be the case for e.g. GC/MS). Previous work has employed standard ^1^H NMR to examine the toxic compounds generated from ENDS use by capturing the vapors in a reservoir while applying vacuum to the e-cigarette with a syringe^14^. Herein, as represented by Fig. 1, we monitor the transition of ENDS liquids in a single cavity using an in situ MAS NMR rotor containing e-liquid, gas, and simulated coil materials to identify the decomposition products of low-temperature electronic cigarette use and explore the decomposition routes by controlling the oxygen availability and the temperature. MAS NMR offers some advantages not available to solution-state NMR. Namely, the presence of solid particles as simulated coil materials offers the ability to investigate the potential interactions between atomizer and e-liquid during the experiment. Further, the heterogeneous nature of the liquid and gaseous overhead phase creates an interface within the magnetic coil which produces magnetic susceptibility-induced spectral broadening when trying to observe both phases at the same time, a capability that can be achieved with this method. Without the use of MAS, the fine line features would not be visible due to signal broadening. Though the exact conditions of an atomizer are not matched due to challenges imposed by metallic heating coils, low conversion from small contact times, low sample quantities, and thus, sensitivity, these conditions are controlled in a manner to allow for in situ comparison between specified conditions, rather than an operando alternative. This strategy, illustrated in Fig. 2 showing the sample and vapor head space, will assist in understanding the potential toxins present in ENDS aerosols generated at low temperatures.

## Results and discussion

### Thermal stability under anaerobic conditions

**Figure 3:**
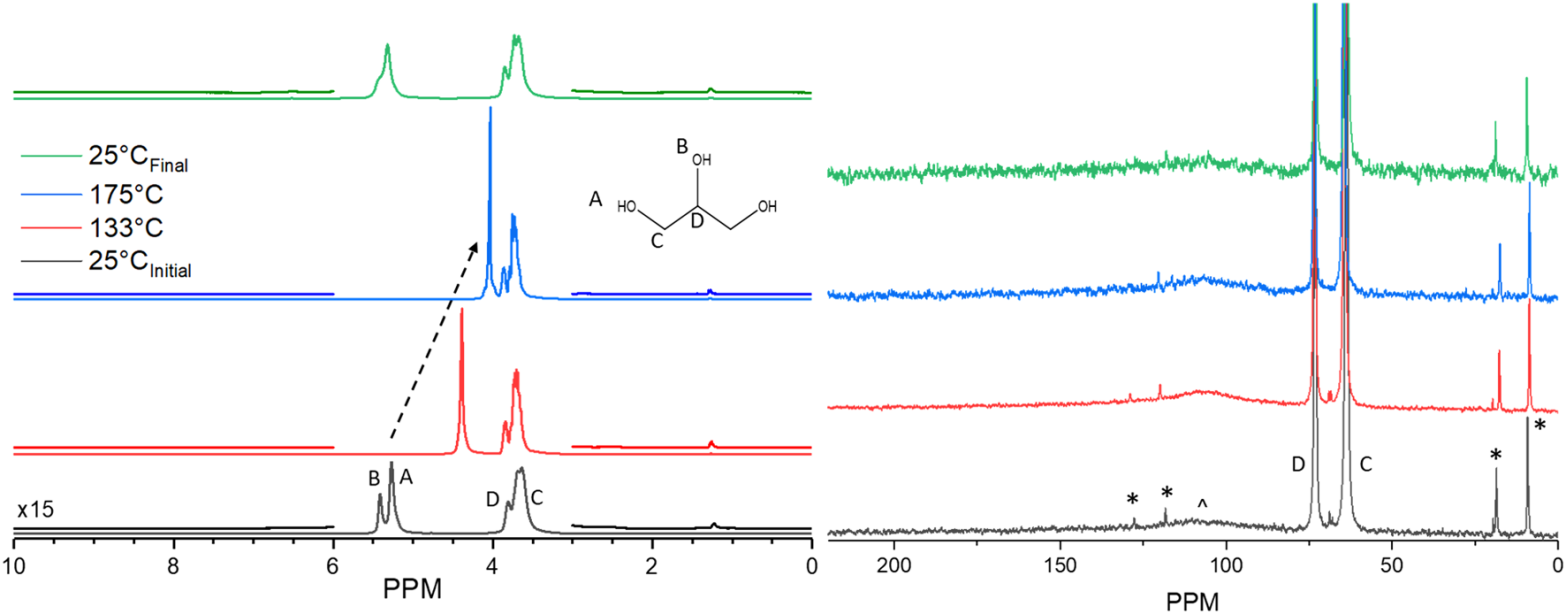
^1^H (left) and ^13^C (right) MAS NMR of 100 μl of glycerol in N_2_ heated to 175 °C. No evidence of thermal decomposition was observed. *indicates spinning sidebands. ^indicates probe background. ^1^H: the spectrum at 133 °C completed after 1 h of heating, 175 °C completed after 1 h of heating at 175 °C. ^13^C: the spectrum at 133 °C completed after 54 h of heating, 175 °C completed after 7 h of heating at 175 °C. The total heating time including temperature ramping was ~ 61 h.

Simulated ENDS liquids were assessed for their low-temperature thermal decomposition under nitrogen atmosphere to consider routes of degradation which may occur in the absence of atmospheric oxygen during the use of ENDS. Previous reports have shown the potential for anaerobic degradation at moderate temperatures (~ 400 °C)^15^, however such conversions at low temperatures of less than 256 °C have not been reported in the field of E-cigarettes. Figure 3 presents the results of an investigation into the potential low-temperature anaerobic conversion of glycerol. The left plot follows the evolution of glycerol-originated ^1^H species present in the NMR sample as a function of experimental temperature. The initial 25 °C spectrum showcases a pair of resonance features at 5.3 and 5.4 ppm which correspond to the alcohol protons on the primary (A, 2 per glycerol) and secondary carbons (B, one per glycerol), respectively. Features at 3.6 and 3.7 ppm represent the methylene protons (C, 4 per glycerol) which inherit slightly modulated chemical environments based on their spatial position relative to the secondary alcohol. The 3.8 ppm peak represents the C-H proton (D, one per glycerol). A very small feature at 1.26 ppm is also present from solution impurities which narrows at elevated temperatures and remains unidentified. At elevated temperatures, both alcohol proton groups migrate upfield to 4 ppm due to reduction in hydrogen bond strength instigated by enhanced thermal motion. No new proton features develop which would indicate the anaerobic conversion of glycerol to a new species. Upon returning the sample to room temperature (25 °C), after nearly two days of heating at 175 °C, the sample retained the features initially present and the alcohol groups migrated to their original position, signifying a return to the initial sample temperature and hydrogen bonding state.

To validate this observation, ^13^C NMR was employed on the same system, alternating measurements between proton and carbon to acquire data at comparable conditions. The spectra in the right side of Fig. 3 show ^13^C resonances at 63 and 73 ppm, corresponding to secondary (D, one per glycerol) and primary (C, two per glycerol) carbons, respectively. Two sets of spinning sidebands (*) as well as a broad probe background (^) accompany the resonances of glycerol. Heating to 175 °C does not generate any new carbon features, confirming the result of the ^1^H NMR data that anaerobic conditions do not stimulate the conversion of simulated ENDS liquids at low temperatures.

### Oxidative thermal decomposition

In the presence of an oxidizing agent, the alcohols of ENDS may transition into new species at elevated temperatures through oxidation. For glycerol, this has been proposed from e-cigarette and sugar chemistry literature to take the form of a cascade of reactions which terminate as toxic carbonyl-containing products. Figure S6 illustrates the commonly proposed pathway for the thermal degradation of glycerol. Initially, dehydration of the alcohol is proposed to form glycidol, which may then undergo a transition to 3-hydroxypropanal which dehydrates to form acrolein and subsequently oxidize to acrylic acid or may directly decompose to formaldehyde and acetaldehyde. Glycerol has also been proposed to dehydrogenate to form either 2,3-diydroxypropanal, 1,3-dihydroxypropan-2-one, or methylglyoxal via glycol. Given the apparent absence of such dehydration steps to generate a stable epoxide (accompanied by transitions to 3-hydroxypropanal and acrolein) in the anaerobic trial from this work (Fig. 1), a contrasting pathway is presumed for low temperature transitions under oxidative conditions.

**Figure 4:**
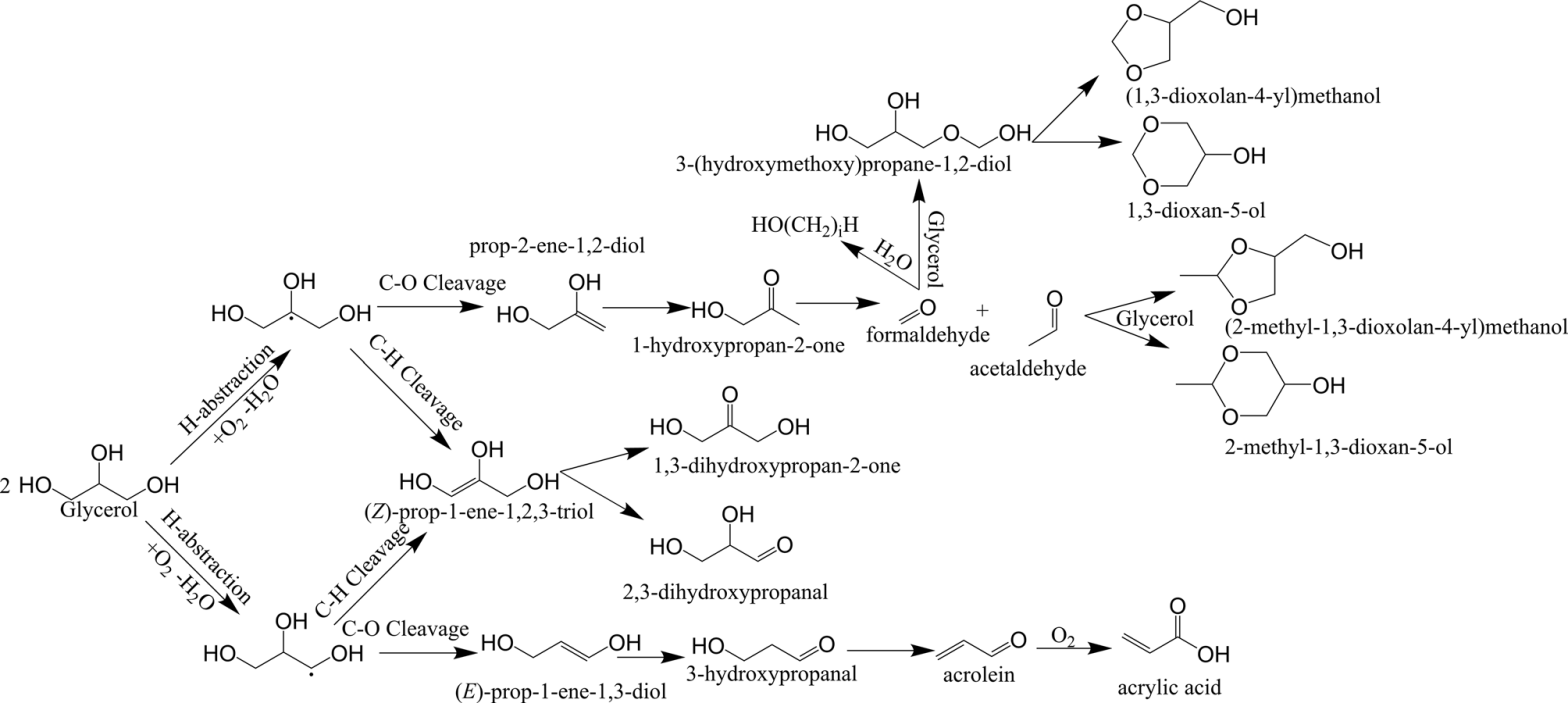
Radical-mediated mechanism for the oxidative degradation of glycerol.

The presence of oxygen clearly enables chemical transitions not possible under inert atmospheres based on the previously disclosed work and the oxidation steps presumed^15^. In line with this, biomass conversion studies have confirmed the ability for oxygen to unlock an entirely new network of chemical conversions which proceed through a low temperature pathway^18^. In this, oxygen may be inserted into the glycerol molecule and generate radical species via H-abstraction (see adapted scheme Figure S7)^14,19^. Figure 4 illustrates the proposed degradation which is initiated by H-abstraction to form an oxy-alkyl radical and reflects the overall balance of oxygen consumption and water generation. The alpha radical may undergo C-O cleavage to generate 1-propene-1,3-diol which tautomerizes to form 3-hydroxypropanal. The subsequent decomposition from 3-hydroxypropanal to acrolein is facile^18^. C–O cleavage of beta-oxy-alkyl radicals leads to instead to 2-propene-1,2-diol which tautomerizes to form 1-hydroxyporan-2-one. Subsequent C–C cleavage generates carbonyl compounds formaldehyde and acetaldehyde. Their successive conversion will be discussed below. Both radical species can also undergo C-H cleavage to form propanetriol, which can tautomerize to either 1,3-dihydroxypropan-2-one or 2,3-dihydroxypropanal.

**Figure 5:**
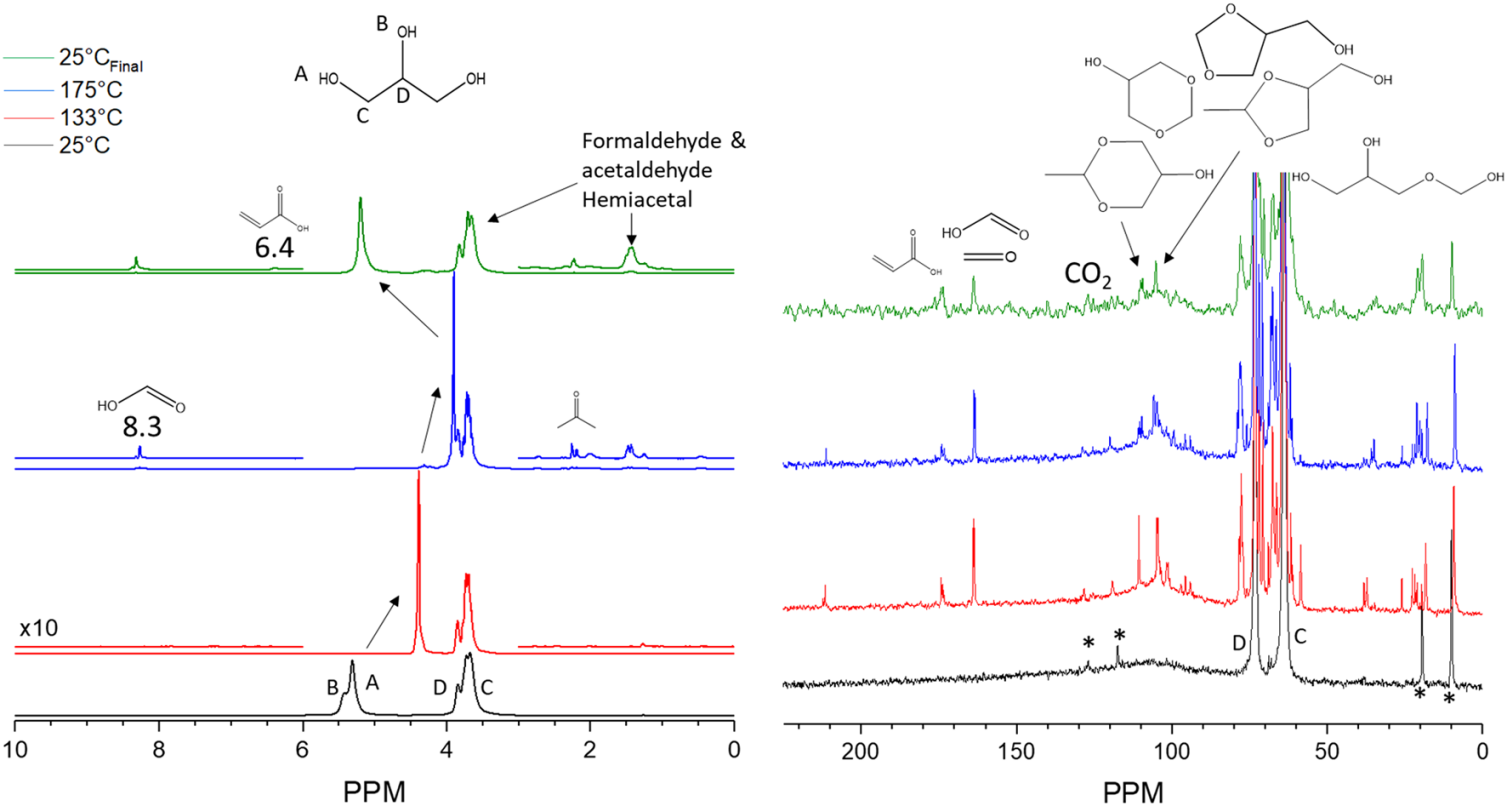
^1^H (left) and ^13^C (right) MAS NMR spectra of 100 μl glycerol in 75 psig O_2_ heated to various temperatures prior to cooling to monitor the thermal degradation processes taking place. ^1^H: the spectrum at 133 °C completed after 1 h of heating, 175 °C completed after 3 h of heating at 175 °C. ^13^C: the spectrum at 133 °C completed after 46 h of heating, 175 °C completed after 29 h of heating at 175 °C. The total heating time including temperature ramping was ~ 77 h.

The thermal degradation of glycerol under oxidative conditions was examined with ^1^H and ^13^C MAS NMR. The proton spectra (Fig. 5, left) reflect similar observations to the stable, N_2_ atmosphere experiment with regards to the major features. Unlike, the previous condition, however, vertically expanding the proton data intensity reveals that numerous new features develop at elevated temperatures. This change is stimulated by the presence of oxygen which reacts with glycerol to generate new compounds. Figure S8 illustrates the dependence of oxygen availability by modulating the oxygen pressure from 1 to 15 psig, showing a greater quantity of products at progressively higher pressures. Indeed, even more are present in this 75 psig O_2_ sample, which represents an O_gas_/C ratio of 0.02. Such a trend establishes that conversion of ENDS liquids is limited by oxygen availability. To guide assignments of the observed NMR peaks, predictions of chemical shifts using ChemNMR (CambridgeSoft) are presented in Table S3. Notably, several peaks form around 1.4, 2.1, possibly ~ 4, and 6.4 ppm. When combined with the results from the ^13^C NMR data, (Fig. 5, right), assignment of these new features becomes possible.

The most apparent features are the array of signals that form between 50 and 80 ppm in the ^13^C spectra at elevated temperatures. These numerous signals exhibit significant signal overlap indicating the similar chemical environment of these carbon atoms. Due to the presence of signals also ~ 100 ppm and new features around 20 ppm, these are assigned to a variety of acetal species. Such species are known to readily form from short-chain aldehydes to generate new hemi-acetal species^20–24^. In water, for example, formaldehyde will combine with water to exothermically generate methylene glycol^25,26^. This can undergo subsequent additions to itself to generate n-methyl glycol, releasing water from the newly oligomerized methylene glycol according to the scheme below:

$${\text{CH}}_{{2}} {\text{O }} + {\text{ H}}_{{2}} {\text{O}} \to {\text{HOCH}}_{{2}} {\text{OH}}$$

$${\text{HO}}\left( {{\text{CH}}_{{2}} {\text{O}}} \right)_{{\text{i}}} {\text{H }} + {\text{ HOCH}}_{{2}} {\text{OH}} \leftrightarrow {\text{HO}}\left( {{\text{CH}}_{{2}} {\text{O}}} \right)_{{{\text{i}} + {1}}} {\text{H }} + {\text{ H}}_{{2}} {\text{O}}$$

Such species readily form with water (Fig. 4, Figure S10, and Figure S11 show formalin) and similar transformations have been reported for alcohols, such as the infrared study by Silverman which demonstrated methoxymethanol from a mixture of formaldehyde and methanol^27^. Dehydration is apparent based on the observation of gaseous water pressure (0.5 ppm) at high temperatures and liquid water at room temperature (4.8 ppm). In the case of glycerol, the probable conversions to hemiacetal and acetals are presented in Figure S12. Indeed, such formations well-represent the observed chemical shifts, enabling the assignments of such features to an array of hemiacetals and acetals generated by glycerol combination with formaldehyde and acetaldehyde as well as methylene glycol from water. At low temperatures, these species exhibit relatively narrow linewidths, but broadening occurs at higher conversions, indicating a range of species with very similar chemical shifts as addition occurs. Such oligomerization products may also explain the presence of a dark, gel-like substance present in the NMR rotor at the end of experiments in which hemi-acetals were present. There may additionally be a trace of gaseous formaldehyde present at ~ 164 ppm, however, formic acid may also contribute to this signal. No such trace was observed for either liquid or gaseous acetaldehyde (Figure S13 and Figure S14), which could be explained by the lower vapor pressure of the two-carbon aldehyde. The presence of these compounds directly suggests that at low temperatures in the NMR rotor, the alpha-oxy-alkyl radical of glycerol undergoes C-O cleavage and further decomposition to form aldehydes. In addition to this decomposition pathway, the C-O cleavage pathway of the beta radical to acrolein is supported as well. The evidence from this stems from the observation of a ^1^H feature at 6.4 and a ^13^C feature at 175 ppm which likely belong to acrylic acid obtained from the oxidation of acrolein, another toxin which may be present in aerosols of ENDS liquids. Oxidation of acrolein to acrylic acid has been reported at higher temperatures^14^, and also in the presence of water over molybdenum-vanadium oxide catalysts at low temperatures^18^. Acrolein itself was not observed in these experiments (Figure S17 and Figure S18), but this represents the first known observation of such species from ENDS liquids at low temperatures without the addition of a catalyst to stimulate the oxidation. No evidence for C-H cleavage pathways (9.7 ppm for 2,3-dihydroxypropanal or 4.7 ppm for 1,3-dihydroxypropan-2-one, analyzed at high temperatures due to signal overlap at low temperatures) exists, signifying the dominance of C-O cleavage pathways. Finally, a peak at ~ 125 ppm may be present in this sample which is indicative of carbon dioxide formation from complete oxidation, potentially from formaldehyde or acrolein. Higher O_gas_/C ratios stimulate the more selective formation of CO_2_ (Figure S9) potentially as the higher temperatures decompose the hemiacetals and enable the released formaldehyde to oxidize.

In addition to the decomposition of glycerol, the second major ENDS component, propylene glycol, has been shown to degrade upon exposure to high temperatures and oxygen. In a similar fashion to glycerol, this molecule was probed in this work to assess its low-temperature thermal degradation. Previously proposed routes include its dehydration to propylene oxide followed by the subsequent decomposition to 2-propeneol or acetone. It could also oxidize and dehydrate to generate acetaldehyde, formaldehyde, and acetone or undergo dehydrogenation to methylglyoxal. Surface-mediated routes have proposed the formation of formaldehyde, acetaldehyde, and methylglyoxal. These high-temperature transitions are depicted in Figure S15.

**Figure 6:**
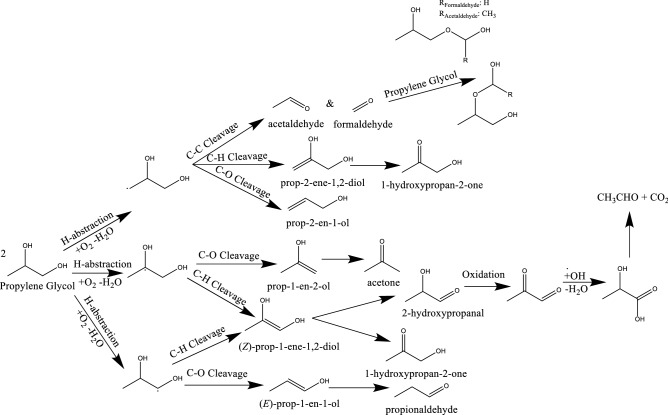
Proposed low-temperature oxidative degradation scheme for propylene glycol.

Given the similarities to glycerol, propylene glycol was also expected to undergo a radical-mediated decomposition mechanism at low temperatures, facilitated by the insertion of oxygen leading to H-abstraction (Fig. 6). The pathway to produce these radicals is presented in Figure S16^14^. After the generation of the alpha-oxy-alkyl radical, C-O cleavage and tautomerization lead to the formation of propionaldehyde. Beta-oxy-alkyl radicals may similarly cleave the C-O bond, which would generate acetone. C-H cleavage of either radical leads to the formation of prop-1-ene-1,2-diol which tautomerizes to 1-hydroxypropan-2-one or 2-hydroxypropanal, and subsequently oxidizes to form formaldehyde and CO_2_. The final radical presents three potential pathways: 1) C-O cleavage to prop-2-en-2-ol, 2) C-H cleavage to 1-hydroxypropan-2-one, and 3) C–C cleavage to aldehydes which may generate acetal species. To discriminate the pathways in operation during low-temperature heating of ENDS liquids, in situ NMR was applied to the oxidative propylene glycol system as well.

**Figure 7:**
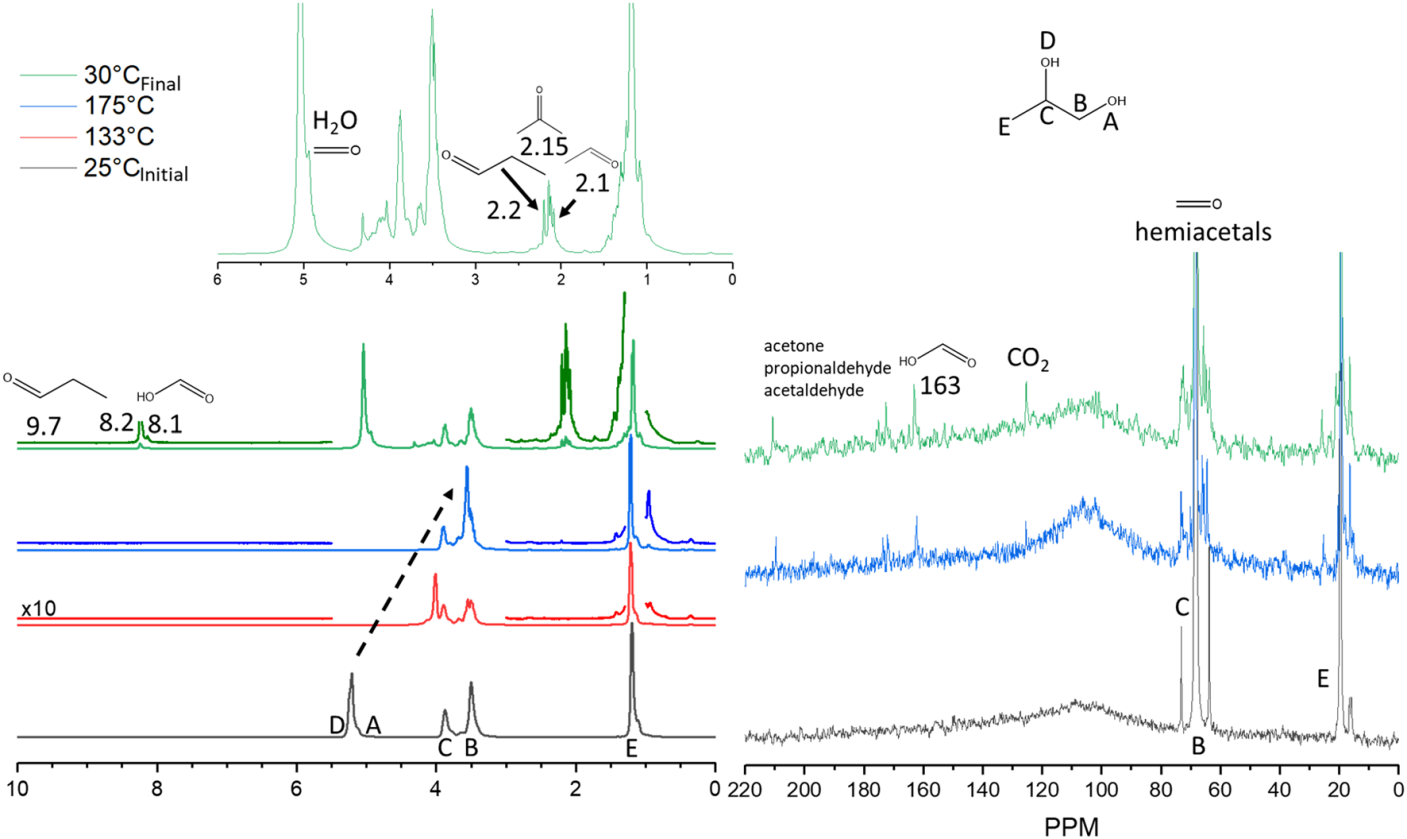
^1^H (left) and ^13^C (right) MAS NMR spectra of 20 μl propylene glycol in 75 psig O_2_ heated to various temperatures prior to cooling to monitor the thermal degradation processes taking place. ^1^H: the spectrum at 133 °C completed after 3.4 h of heating, 175 °C completed after 3.4 h of heating at 175 °C. ^13^C: the spectrum at 175 °C completed after 19.8 h of heating at 175 °C. The total heating time including temperature ramping was ~ 24 h.

Figure 7 shows the results for the ^1^H and ^13^C experiment to determine the oxidative decomposition routes of propylene glycol. At room temperature, alcohol protons (A and D) can be seen at ~ 5.1 ppm. The C-H group on the second carbon position is observed at ~ 3.8 ppm (C), the CH_2_ at 3.4 ppm (B), and the methyl group (E) at ~ 1.2 ppm. As the temperature increased, the alcohol protons shifted upfield to ~ 3.5 ppm, a process that was reversible upon decreasing to room temperature. Throughout this process, several species formed. The feature at 9.7 ppm may indicate the presence of propionaldehyde, further evidenced by a peak at 2.2 ppm and previous assignment^14^. Distinctive due to their separation from other resonance features were a pair of peaks at 8.1 and 8.2 ppm. The feature at 8.1 ppm may correspond to the formation of formic acid through the oxidation of formaldehyde. This is strengthened by the appearance of a feature at 163 ppm in the ^13^C spectrum and solidified by its previous assignment^14^. The second peak remains unassigned.

Two additional features are present at 2.15 and 2.1 ppm, which may correspond to acetone and acetaldehyde formation. Another peak at ~ 4.8 ppm likely come from water generated during dehydration but is at a similar position to that observed with the formaldehyde-water mixture in the supporting information. Closer inspection of the ^13^C NMR data reveals that CO_2_ is indeed formed under this O_gas_/C ratio of 0.15, indicating more complete oxidation has occurred. Further, numerous peaks from hemiacetals are observed between 60 and 80 ppm in the ^13^C NMR spectra. These results indicate that low-temperature thermal degradation may produce toxic species such as formaldehyde and acetaldehyde and their derivatives as well as formic acid. The absence of methylglyoxal in all experiments conducted at low temperature further supports these proposed radical-mediated pathways under these conditions since they have been observed at higher temperatures via contrasting mechanisms. In this mechanism, the absence of C-O cleavage to prop-2-en-2-ol and C-H cleavage to 1-hydroxypropan-2-one suggest these pathways are not active under these conditions, however, other cleavage pathways cannot be ruled out given the final products observed.

### Solution mixtures of propylene glycol and glycerol

**Figure 8:**
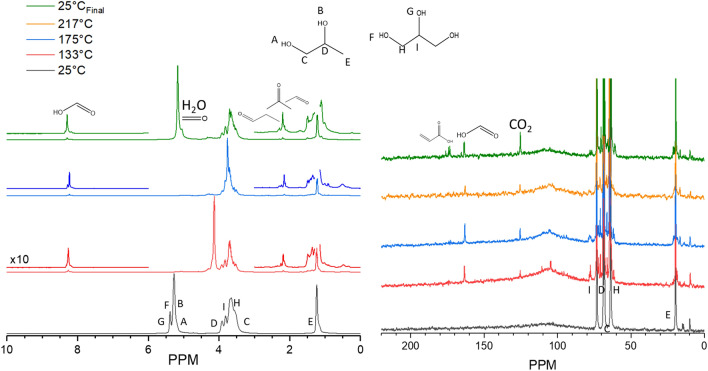
^1^H (left) and ^13^C (right) MAS NMR spectra of a mixture of 20 μl propylene glycol and 20 μl glycerol in 75 psig O_2_ heated to various temperatures prior to cooling to monitor the thermal degradation processes taking place. ^1^H: the spectrum at 133 °C completed after 48 h of heating, 175 °C completed after 39 h of heating at 175 °C, 217 °C completed after 7.8 h of heating at 217 °C. ^13^C: the spectrum at 133 °C completed after 42 h of heating, 175 °C completed after 36 h of heating at 175 °C, 217 °C completed after 5 h of heating at 217 °C. The total heating time including temperature ramping was ~ 150 h.

Mixtures of the two constituents (propylene glycol and glycerol with equal volume) are the primary ingredients to ENDS liquids. As such, their thermal decomposition behavior together is of interest to assessing the toxic compounds produced during low-temperature electronic cigarette use. Figure 8 documents the findings of such a trial. A very similar array of species is observed in this sample as to the individual component trials. Significant overlap of the -OH and C-H protons in the ^1^H NMR exists between the two species, complicating their distinct observation, however, labels A-I have been placed to denote the appropriate position. The presence of water indicates that these low temperature, radical-mediated dehydration routes are active in these samples (4.8 ppm at room temperature, 0.5 ppm in the gaseous phase). Additional features previously assigned for acetone, and acetaldehyde were observed as well and numerous methyl signals around 1.2 ppm. In the ^13^C NMR spectra, an abundance of CO_2_ (125 ppm) was observed in addition to peaks ascribed to both formic (163 ppm) and acrylic acid (172 ppm) at room temperature. Hemi- and formal acetals were also observed, evidenced by numerous resonance features between 60 and 80 ppm. Some species at ~ 100 ppm were present at 133 °C, but based on their absence/reduction in signal intensity, appear to have converted to other products, potentially CO_2_, at higher temperatures such as 217 °C. The dissipating features near 100 and 110 ppm are indicative of acetaldehyde-derived acetals from glycerol degradation. Such an observation highlights the importance of observing these transitions and speciation at temperatures which represent the puffing regime of interest, in this case direct liquid contact to the coil. The results show that in mixtures, both the glycerol and propylene glycol contribute to the thermally degraded products and can both generate toxic aldehydes. What is unique in this condition, however, is the rate at which these transitions occur. Figure S20 provides a comparison of each component alone combined at different temporal positions and shows that the mixture with the reaction is slower than the individual components. In the glycerol case, the sample has reached an equilibrium after just 36 h at 150 °C. The mixture, however, continues degrading up to at least 135 h. Propylene glycol transitions are presented for the available time points at 150 °C and 200 °C and qualitatively suggest that the mixture is slower than this individual component as well. A portion of this may be explained by the lower O_gas_/C ratio of 0.07 (compared to 0.15) since O_2_ is necessary for even the initial stages of conversion via the radical-mediated mechanism. The rate of these transitions may well be second order with respect to oxygen (rate potentially ~ 4 times as fast with twice the oxygen content), but confirmation is a topic for future endeavors. This is a strong indicator that the oxygen flow into the atomizer may direct the extent to which ENDS liquids degrade and a deep understanding of these topics may improve the safety of ENDS use.

### Effect of catalytic surfaces

It has been well-documented that at higher temperatures, the surface of an ENDS coil enhances the extent to which thermochemical conversion of the ENDS liquids takes place. The same concept was investigated herein by preparing metal oxides Cr_2_O_3_ and ZrO_2_ as substitutes for an ENDS coil. The oxides of the parent coil materials were selected to represent the passivation layer of the coil which would interact with the organic substrates. Stainless-steel powder was considered but exhibited trace magnetic properties which prevented NMR measurements. To assess potential anaerobic, catalyzed pathways, Cr_2_O_3_ was heated in the rotor with the propylene glycol and glycerol mixture for over 40 h (Figure S20) at temperature up to 220 °C. No significant changes in the spectra were observed as in the N_2_ atmosphere case without a catalyst surface. Oxygen was introduced followed by the same heating procedure and spectra were monitored for degradation (Figure S21). This yielded similar observed species to the catalyst-absent sample, though apparently quantitively fewer. It should be noted that such a lack of detection may be due to signal broadening induced by the surface, however, gaseous products should be readily observable and even CO_2_ detection was low. This result shows an inhibiting effect of material surfaces on ENDS liquid degradation at low temperatures which proceeds via a radical-mediated mechanism.

**Figure 9:**
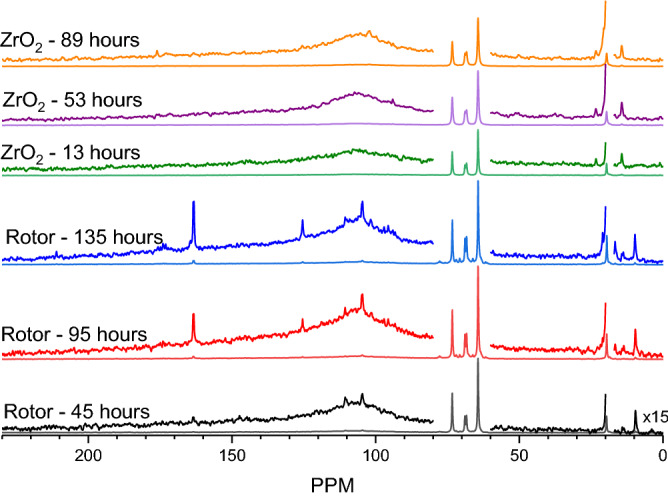
^13^C MAS NMR spectra of 20 μl propylene glycol and 20 μl glycerol under 75 psig O_2_ with and without 51.3 mg ZrO_2_ at different reaction times. The spectra were collected at 133 °C.

Since ZrO_2_ is a well-known acid–base catalyst and this material also represents the material of construction for the NMR rotor, it was used as the solid powder catalyst to examine the potential role of ZrO_2_ rotor in the conversion of ENDS liquids. The results are comparable to the Cr_2_O_3_-containing experiment except for intense features from 1 to 1.5 ppm in the ZrO_2_ sample. Kinetic comparison of the ZrO_2_-containing sample to the rotor blank are presented in Fig. 9. These represent the ^13^C NMR data for the respective samples containing the same quantity of ENDS liquids and the same 75 psig O_2_ pressure (O_gas_/C = 0.07) at different reaction times. While some products are visible for the rotor-only experiment at 45 h, these only begin to appear at 89 h for the ZrO_2_-containing sample. This result suggests that the ZrO_2_ in the rotor is not appreciably contributing to the conversion of the ENDS liquid mixture since such a dramatic increase in surface area did not enhance the reaction rate, supporting the radical-mediated mechanism. In contrast, its presence apparently retards conversion, potentially due to surface interactions which stabilize the chemical species and prevent oxygen insertion. The consistency between Cr_2_O_3_ and ZrO_2_ catalyst containing samples suggests that the low temperature pathway is not strongly dependent on the presence of a material surface. The detailed underlying mechanisms of propylene glycol and glycerol on catalyst surfaces may assist in understanding the chemistry taking place in the ENDS atomizer and represents a topic for future surface science investigation.

The data presented herein help to identify the decomposition of ENDS liquids and show that toxic compounds are produced which depend strongly on the oxygen availability at these low temperatures. It should be noted that these data were collected over long periods of time due to the poor sensitivity of the NMR technique, rendering more advanced NMR methods such as 2D TOCSY, ^1^H-^13^C HETCOR, COSY, HMBC, and HSQC which would help provide evidence of species identification very challenging. Collecting a single 2D ^1^H spectra would require on the order of one day given the low quantities of substrate used. Further, the dynamic process at higher temperatures would impart noise to the data as the sample changed over time, limiting them to spectra collected at the end of each experiment. Fast 2D methods, such as SOFAST, might play a future role in investigating ENDS liquids^28^. ^13^C isotopic labeling would also help to expedite the data collection process, enabling better temporal resolution and should be regarded as strategies for future studies.

## Conclusion

**Figure 10:**
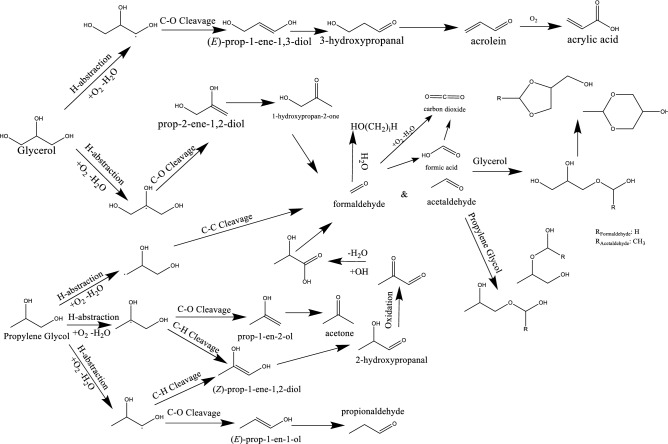
Chemical transformation pathway for low-temperature, liquid phase ENDS liquids.

Herein, we have examined the low-temperature thermal degradation of propylene glycol and glycerol, the primary constituents of ENDS liquids. Monitoring the low-temperature degradation pathway over an extended period of time has enabled the detection of converted chemical species by natural abundance ^13^C NMR and ^1^H NMR which may well represent the types of species present in ENDS vapors and aerosols at these temperatures. The results demonstrate that the degradation of ENDS liquids is strongly dependent on the oxygen availability, both in the presence and absence of a catalyst surface, evidenced by the strong effect of the O_gas_/C ratio. When oxygen is available, the ENDS liquids decompose at temperatures below 200 °C and form numerous chemical species via a radical-mediated mechanism initiated by molecular oxygen. Among the species formed, formic and acrylic acids are generated which represent health risks upon inhalation^29,30^. Further, the formation of hemi- and formal acetals is abundant, signifying the generation of both formaldehyde and acetaldehyde in ENDS aerosols. The dangers of formaldehyde in heated nicotine products has been well-established^31^, though subsequent discussion has suggested that such dangers in e-cigarettes are over-stated. These new results show clearly the presence of such aldehydes in ENDS liquids thermally degraded at low temperatures. Interestingly, simulated ENDS coil surfaces do not act as catalysts in the low temperature regime indicative of a liquid saturated coil, evidenced by the lack of apparent increase in decomposition rate, potentially due to strong surface binding which prevents conversion. The radical-mediated mechanism is the most likely explanation for the absence of any conversion (including dehydration) when oxygen is not present. The observed species and their likely routes have been summarized in Fig. 10. Observation of intermediate species to confirm this pathway could manifest from electron paramagnetic resonance (EPR) spectroscopy to directly observe ENDS radicals as they form or via isotopic labeling of ^13^C in the parent alcohols to enhance the spectral sensitivity, and thus temporal resolution. Enhancing the sensitivity by ~ 90 times, for example, would potentially enable the observation of the relatively short-lived non-radical intermediate species which convert too quickly under current conditions, solidifying the active steps in the pathway. The results outlined herein highlight the conversion of ENDS at low temperatures to form toxic compounds which may potentially be inhaled in significant quantities over extended low-temperature ENDS device use.

## Methods

In situ ^1^H and ^13^C MAS NMR spectroscopy measurements were conducted on a Varian-Agilent Inova wide-bore 300 MHz NMR spectrometer using a commercial 7.5 mm ceramic pencil type MAS probe, operating at a ^1^H and ^13^C Larmor frequencies of 299.969 MHz and 75.430, respectively. Samples were rotated at the magic angle, spinning at ~ 3.5 kHz, which enabled enhanced resolution in these primarily liquid systems (Figure S1). Single pulse ^1^H (^13^C) sequences consisting of a π/4 pulse width of 2.25 μs (3.5 μs), acquisition time of 999 ms (400 ms), and recycle delay of 5 s (4 s). Typically, 2048 (32,000) repetitions were used to collect the spectra. All spectra were externally referenced to adamantane at 1.82 ppm and 38.48 ppm for ^1^H and ^13^C, respectively. The experimental temperature was calibrated with an external ethylene glycol thermometer (Figure S2) and temperature-dependent chemical shifts were assessed as previously explored^32,33^. Given that the experimental temperatures employed are near the boiling points of the primary constituents, the vapor pressures have been plotted for the reader’s reference, where Antoine’s constants have been extracted from Perry’s Handbook (Table S1 and Figure S3-S5)^34^. Given the rotor dimensions, it is expected that only a minority of the alcohol species are vaporized during the experiments. The sealed nature of the system dictates that at elevated temperatures, the pressure will increase. Table S2 displays the expected system pressure at various conditions.

ZrO_2_ and Cr_2_O_3_ (Sigma) solid samples were prepared by pre-treating the solid powders under vacuum at 350 °C for 4 h. The solid samples were subsequently loaded into sealable NMR rotors in a dry N_2_-filled glove box. Stainless steel powder was also considered, but even the non-magnetic variety exhibited magnetic properties when positioned near the NMR spectrometer, prevent its use in the instrument. Proplyene glycol (Sigma, 99.5%), glycerol (Sigma, 99.9%), 20% ^13^C-formaldehyde (Sigma, water), acetaldehyde (Sigma, 99%), acrolein (Thomas), and O_2_ (Oxarc) were employed for this study. The desired simulated e-cigarette juice formulations were injected into the rotor via micro syringe before the sample cell was sealed^35,36^. The rotor was subsequently transferred to a specially designed loading chamber where either N_2_ or O_2_ gas was added at the specified pressures^37^. Quantities of ENDS liquids were selected to mimic the quantity of vapor evolved in an average puff (~ 10–50 mg)^14,38^. It should be noted that maximum oxygen pressures of 76 psig were employed (~ 1 ml STP), which is dramatically lower than the quantity of oxygen that would pass through the ENDS device (the typical human lung capacity is 4–6 L. At 20% O_2_ in the air, equates to 800 ml of oxygen). As shown herein, oxygen availability strongly correlates to reactivity suggesting that higher oxygen availability in the real condition may mitigate a portion of the disparities in heating time which are longer in the experiments herein.

## Supplementary Information

Supplementary Information.
