# Supplementary material for: Low-temperature (< 200 °C) degradation of electronic nicotine delivery system liquids generates toxic aldehydes
Source: Sci Rep. 2021 Apr 8;11:7800. doi: 10.1038/s41598-021-87044-x (PMC8032854; doi:10.1038/s41598-021-87044-x)
Supplement: Supplementary file 1 — Supplementary Information. [file 41598_2021_87044_MOESM1_ESM.docx]

# Supporting Information for

# Low-temperature (<200°C) degradation of electronic nicotine delivery system liquids generates toxic aldehydes

Nicholas R. Jaegers, Wenda Hu, Thomas J. Weber, and Jian Zhi Hu*

*Pacific Northwest National Laboratory, Richland, WA 99354, USA*


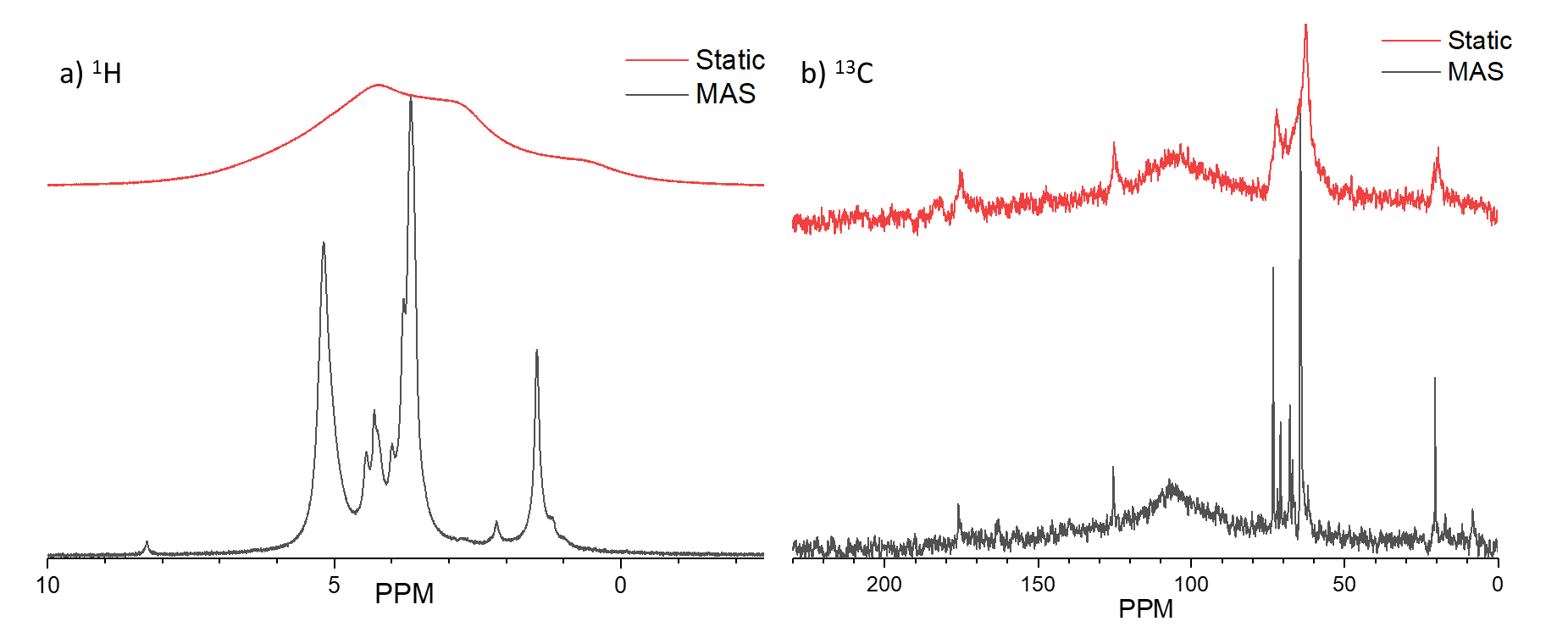


Figure S1. Room temperature ^1^H MAS NMR (a) and ^13^C MAS NMR (b) of 20 μl of glycerol and 75 psig O_2_ reacted over 50.1 mg dehydrated ZrO_2_ at 175°C.


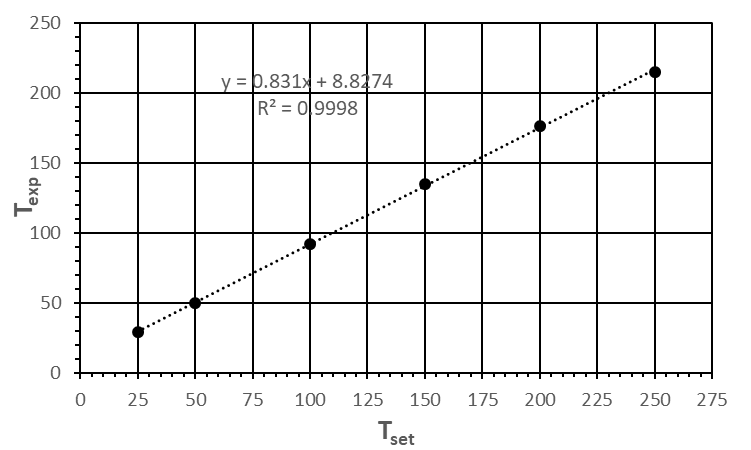


Figure S2. Heating set temperature vs experimental temperature as determined by a 250 μl ethylene glycol thermometer. Temperature expressed in degrees centigrade.

$$P_{s}(Pa(K))=e^{\left[ C1+\frac{C2}{T}+C3*ln\left( T \right)+C4*T^{C5} \right]}$$

Table S1. Antoine equation constants for the vapor pressure of propylene glycol.

| **Chemical** | **C1** | **C2** | **C3** | **C4** | **C5** | **T_min_ (K)** | **T_max_ (K)** |
| --- | --- | --- | --- | --- | --- | --- | --- |
| Propylene Glycol | 212.8 | -15420 | -28.109 | 2.16*10^-5^ | 2 | 213.15 | 626 |


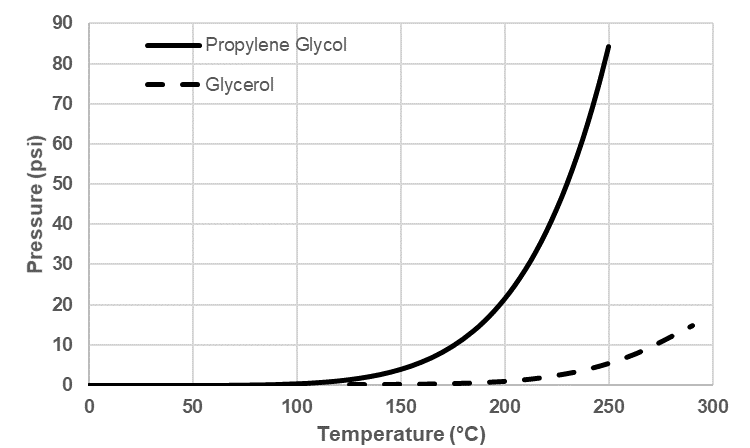


Figure S3. Vapor pressures of propylene glycol and glycerol as a function of temperature.


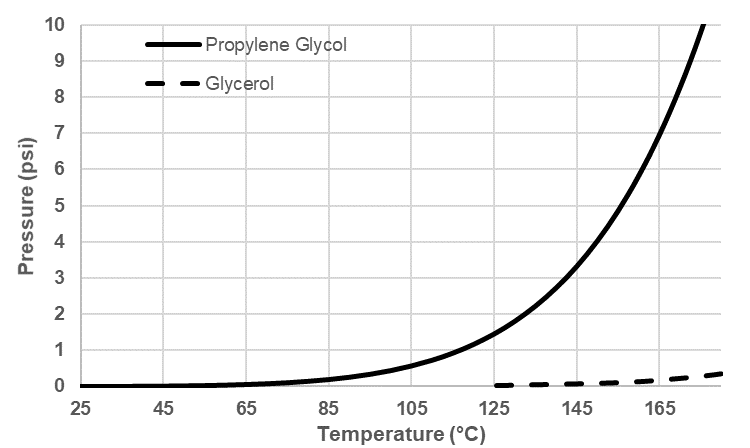


Figure S4. Vapor pressures of propylene glycol and glycerol as a function of temperature.


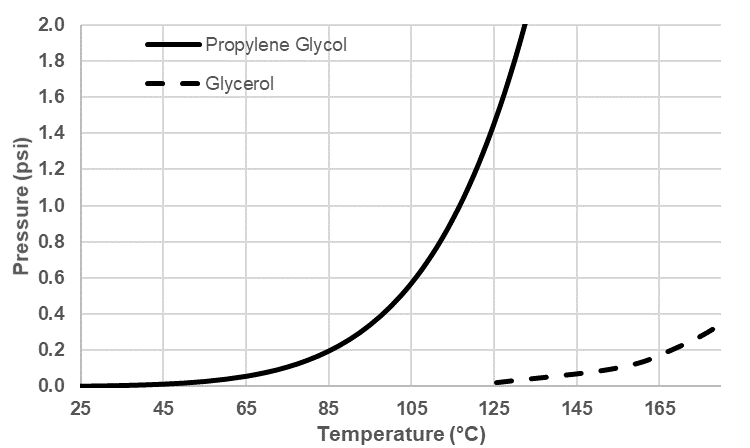


Figure S5. Vapor pressures of propylene glycol and glycerol as a function of temperature.

Table S2. Approximate system pressure at elevated temperatures used in this study.

| **Temperature °C** | **Vapor Pressure Glycerol (psi)** | **Vapor Pressure Propylene Glycol (psi)** | **Gas Pressure (psi)** | **Total Pressure (psi)** |
| --- | --- | --- | --- | --- |
| 25 | ~0 | ~0 | 75 | 75 |
| 133 | ~0.05 | ~1.8 | 102 | 104 |
| 175 | ~0.3 | ~9 | 113 | 122 |
| 217 | ~2 | ~30 | 123 | 155 |

Table S3. Predicted ^1^H and ^13^C chemical shifts of relevant compounds.

| **Structure** | **Chemical** | **Δ ^1^H_calc_** | **Δ ^13^C_calc_** |
| --- | --- | --- | --- |
|  | Acetaldehyde | CH: 9.79  CH_3_: 2.20 | CH: 199.9  CH_3_: 30.7 |
| 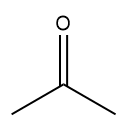 | Acetone | CH_3_: 2.13 | C: 206.4  CH_3_: 30.6 |
|  | Acrolein | OCH: 9.68  CH: 6.27  CH_2_: 6.28 | OC: 194.5  CC: 138.5  CH_2_: 137.6 |
|  | Acrylic Acid | C^1^OH: 12.05  C^2^H: 6.29  C^3^H_2_: 5.99, 6.64 | C^1^OH: 172  C^2^H: 127.5  C^3^H_2_: 134.1 |
|  | Butyraldehyde | OCH: 9.72  C^2^H_2_: 2.38  C^3^H_2_: 1.65  CH_3_: 0.98 | OC: 202.2  C^2^: 45.7  C^3^: 15.5  CH_3_: 13.6 |
|  | CO |  |  |
|  | CO_2_ |  | 124.2 |
|  | 2,3-Dihydroxypropanal | C^1^H: 9.72  C^2^H: 4.46  C^2^OH: 6.18  C^3^H_2_: 4.13, 3.88  C^4^OH: 4.94 | C^1^: 200.8  C^2^: 92.1  C^3^: 59.2 |
|  | 1,3-Dihydroxypropan-2-one | OH: 4.91  CH: 4.69 | C^1,3^: 66.4  C^2^: 201.3 |
|  | (1,3-dioxolan-4-yl)methanol | O_2_CH_2_: 5.4, 5.5  OCH_2_: 3.36, 3.61  OCH: 3.5  OHCH_2_: 3.51, 3.57  COH: 3.94 | O_2_CH_2_: 95.2  OCH_2_: 68.2  OCH: 78.1  OHCH_2_: 61.0 |
|  | (2-methyl-1,3-dioxolan-4-yl)methanol | CH_3_: 1.39  O_2_CH: 3.18  OCH_2_: 3.62, 3.87  OCH: 3.92  OHCH_2_: 3.51, 3.57  COH: 3.94 | CH_3_: 20.6  O_2_CH_2_: 105.3  OCH_2_: 66.7  OCH: 78.0  OHCH_2_: 61.3 |
|  | 1,3-dioxan-5-ol | O_2_CH_2_: 4.87, 4.89  OCH_2_: 3.83, 4.08  CH: 3.78  CHOH: 5.77 | O_2_CH_2_: 94.3  OCH_2_: 71.6  CH: 63.5 |
|  | 2-methyl-1,3-dioxan-5-ol | CH_3_: 1.32  O_2_CH: 4.5  OCH_2_: 3.83, 4.08  CH: 3.78  CHOH: 5.77 | CH_3_: 20.6  O_2_CH: 106.9  OCH_2_: 71.4  CH: 63.8 |
|  | Formaldehyde | 9.6 |  |
|  | Formic Acid | OH: 12.6  CH: 8.05 | 165.7 |
|  | Glycerol | C^1^OH: 3.94  C^1,3^: 3.5  C^2^: 3.06  C^2^OH: 5.77 | C^1,3^: 63.5  C^2^: 74.2 |
|  | Propane-1,1,2,3-tetraol | OH: 4.2, 4.2, 5.8, 3.9  CH: 5.6, 3.6  CH_2_: 3.6, 3.5 | CH: 92.9  CH: 83  CH_2_: 60.1 |
|  | Glycidol | OCH_2_: 2.36, 2.61  CH: 2.54  CH_2_: 3.51, 3.57  OH: 4.94 | OC^1^H_2_: 43.9  C^2^H: 54.5  C^3^H_2_: 63.1 |
|  | Glyoxal | 9.69 | 190 |
|  | 3-hydroxypropanal | OC^1^H: 9.72  C^2^H_2_: 2.59  C^3^H_2_: 3.86  OH: 6.34 | OC^1^H: 202  C^2^H_2_: 45.4  C^3^H_2_: 54.8 |
|  | 1-(hydroxymethoxy)propan-2-ol | OH: 4.12  OH: 5.37  CH_2_: 5.61  CH: 3.9  CH_2_: 3.63, 3.38  CH_3_: 1.11 | CH_2_: 89.5  CH: 67.7  CH_2_: 76.4  CH_3_: 19.8 |
|  | 1-(1-hydroxyethoxy)propan-2-ol | OH: 4.52  OH: 5.37  CH: 5.5  CH: 3.9  CH_2_: 3.63, 3.38  CH_3_: 1.39  CH_3_: 1.11 | CH: 106.8  CH: 68  CH_2_: 73.9  CH_3_: 17.9  CH_3_: 19.8 |
|  | 2-(hydroxymethoxy)propan-1-ol | OH: 4.12  OH: 4.94  CH_2_: 5.61  CH: 3.4  CH_2_: 3.57, 3.51  CH_3_: 1.32 | CH_2_: 86.7  CH: 76  CH: 67.3  CH_3_: 17.3 |
|  | 2-(1-hydroxyethoxy)propan-1-ol | OH: 4.5  OH: 4.9  CH: 5.5  CH: 3.4  CH_3_: 1.32, 13.9  CH_2_: 3.57, 3.51 | CH: 104  CH: 73.5  CH_2_: 67.6  CH_3_: 18.2, 17.6 |
|  | 3-(hydroxymethoxy)propane-1,2-diol | HOC^1^: 3.94  C^1^H_2_: 3.53, 3.59  C^2^OH: 5.77  C^2^H: 3.8  C^3^H_2_: 3.38, 3.68 C^4^H: 5.61  C^4^OH: 4.12 | C^1^H_2_: 63.8  C^2^H: 70.7  C^3^H_2_: 70.2 C^4^H: 89.5 |
|  | 2-hydroxypropanal | OH: 5.48  CH: 9.72  CH: 4.56  CH_3_: 1.26 | CH: 200.8  CH: 85.7  CH_3_: 15.8 |
|  | 1-hydroxypropan-2-one | OH: 4.91  CH_2_: 4.69  CH_3_: 2.24 | C: 203.1  CH_2_: 73.9  CH_3_: 25.6 |
|  | H_2_O | 4.8 |  |
|  | Methylglyoxal | CO: 9.69  CH_3_: 2.17 | C^1^O: 190.0  C^2^O: 196.5  CH_3_: 25.2 |
|  | Prop-1-en-2-ol | OH: 16.77  CH_3_: 1.99  H: 5.1  H: 5.04 | C: 171.5  CH_2_: 70.6  CH_3_: 27.2 |
|  | Prop-1-en-1-ol | OH: 12.58  CH_3_: 2.05  H: 4.85  H: 5.76 | CH: 168.6  CH: 103.4  CH_3_: 12.2 |
|  | Prop-1-en-1,2-diol | OH: 16.77  OH: 12.58  CH_3_: 1.99  H: 4.85 | C: 150.1  CH: 133.3  CH_3_: 20.1 |
|  | 2-Propenol | C^1^H_2_: 5.31, 5.43  C^2^H: 6.07  C^3^H_2_: 4.18  OH: 5.05 | CH_2_: 114.9  CH: 137.5  CH_2_OH: 63.3 |
|  | Propionaldehyde | CO: 9.72  CH_2_: 2.44  CH_3_: 1.04 | CO: 202.2  CH_2_: 37.3  CH_3_: 6.5 |
|  | Propylene Glycol | C^1^OH: 4.94  C^1^H_2_: 3.56  C^2^H: 3.7  C^2^OH: 5.37  CH_3_: 1.11 | C^1^OH: 67.8  C^2^OH: 68.5  C^3^H_3_: 19.5 |
|  | Propylene Oxide | CH_2_: 2.61, 2.36  CH: 2.96  CH_3_: 1.32 | CH_2_: 47.5  CH: 49.8  CH_3_: 19.1 |
|  | Valeraldehyde | C^1^O: 9.72  C^2^H_2_: 2.38  C^3^H_2_: 1.58  C^4^H_2_: 1.38  CH_3_: 0.93 | C^1^O: 202.2  C^2^H_2_: 43.2  C^3^H_2_: 24.3  C^4^H_2_: 22.2  CH_3_: 13.8 |


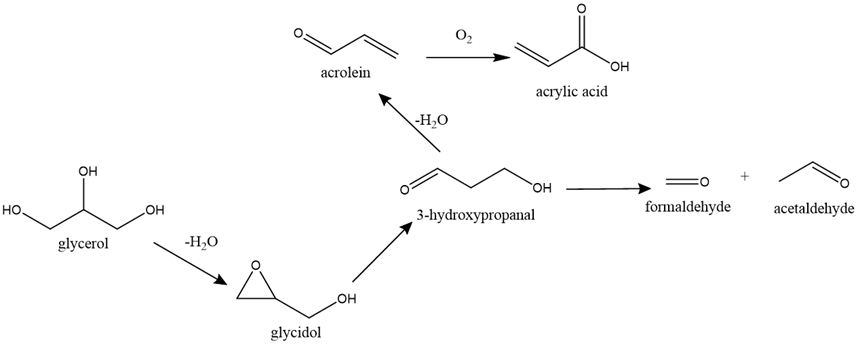


Figure S6. Commonly proposed scheme for the oxidative degradation of glycerol.

Figure S7. Radical formation mechanism for glycerol.


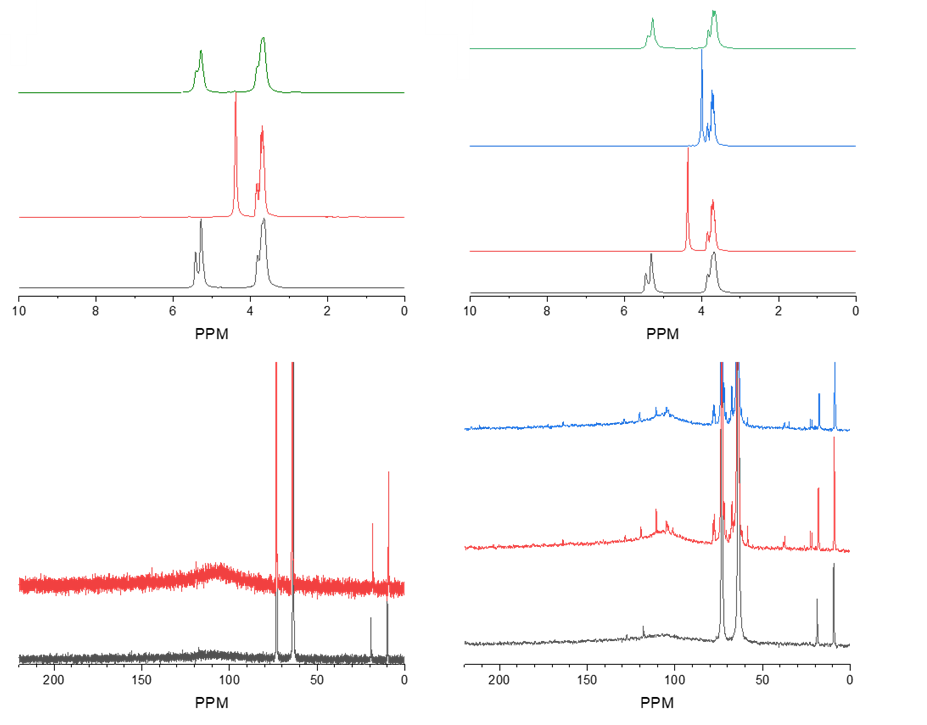


Figure S8. ^1^H (top) and ^13^C (bottom) MAS NMR spectra of 100 μl glycerol in O_2_ heated to various temperatures prior to cooling to monitor the thermal degradation processes taking place. The sepctra represent different pressures of 1(left) and 15 (right) psig.


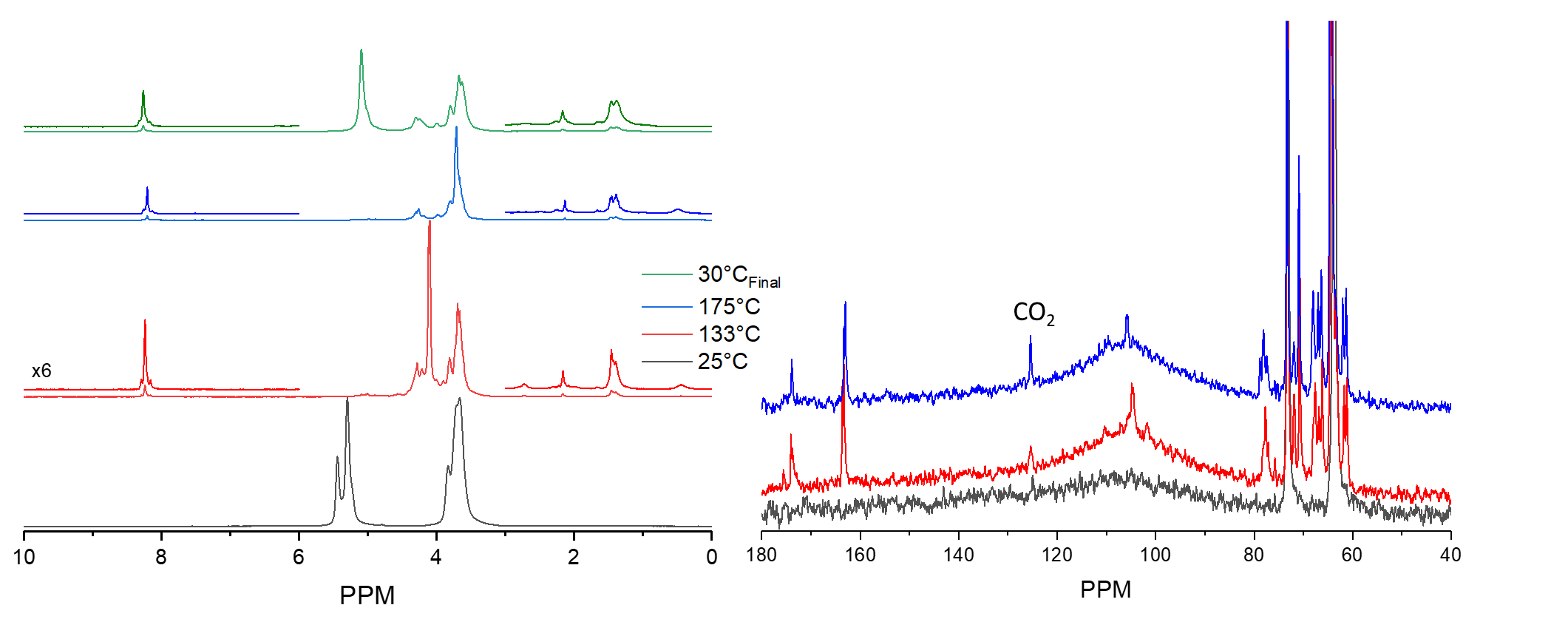


Figure S9. ^1^H (left) and ^13^C (right) MAS NMR spectra of 20 ul glycerol in 75 psig O_2_.


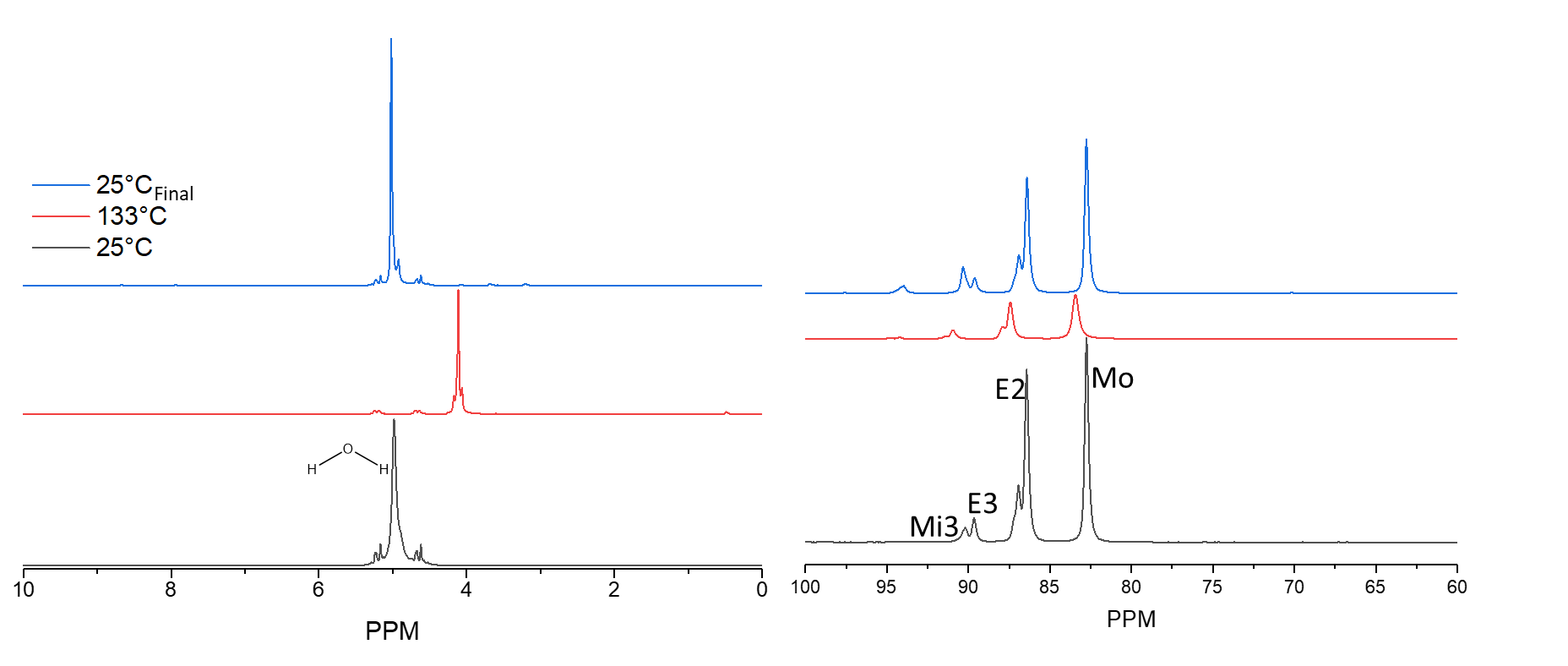


Figure S10. ^1^H (left) and ^13^C (right) MAS NMR spectra of 20 μL 20% formaldehyde (balance water) in N_2_.


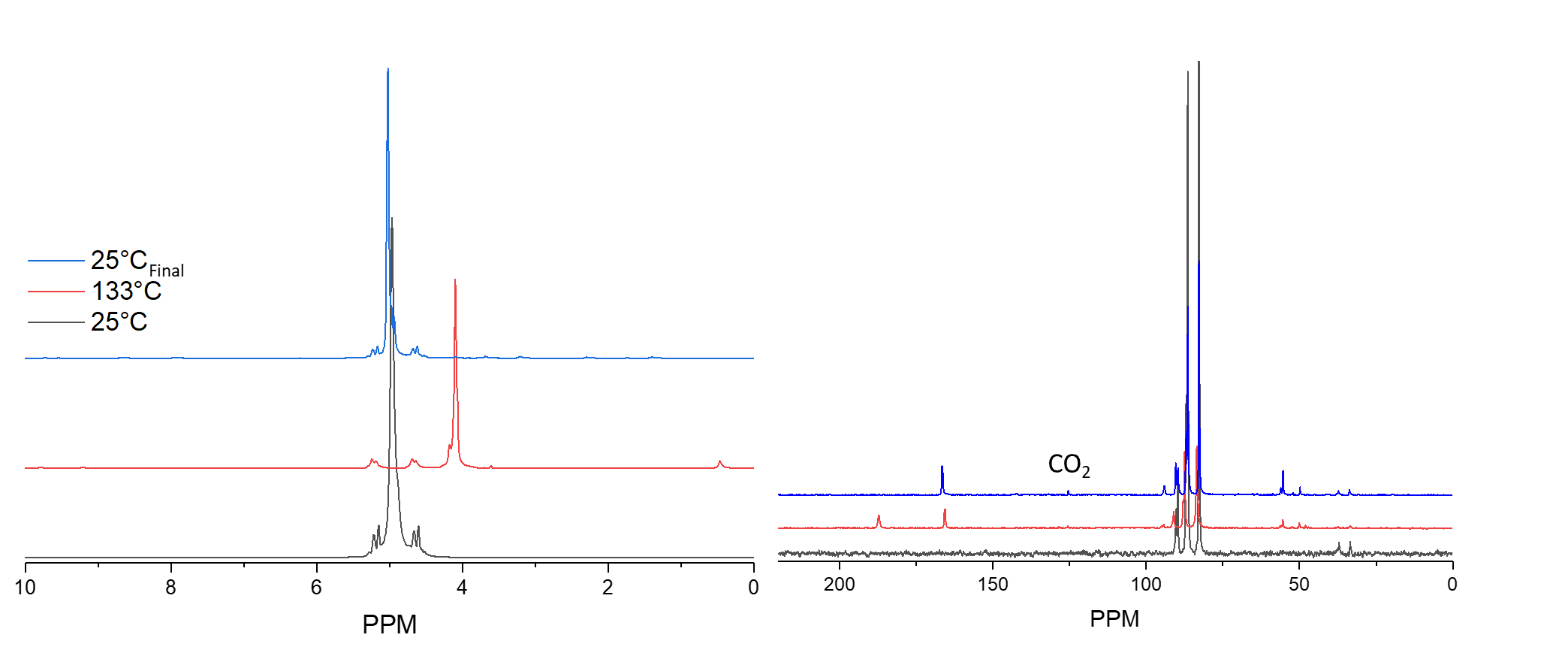


Figure S11. ^1^H (left) and ^13^C (right) MAS NMR spectra of 20 μL 20% formaldehyde (balance water) in 75 psig O_2_.


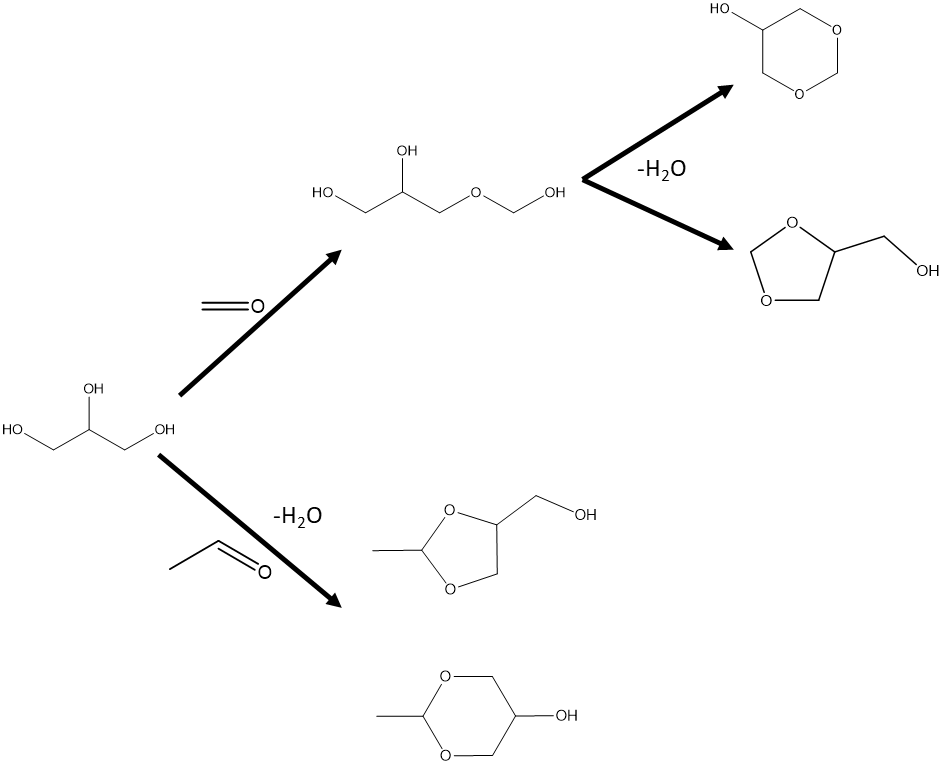


Figure S12. Scheme for acetal formation from water (top) and glycerol with formaldehyde or acetaldehyde (bottom).


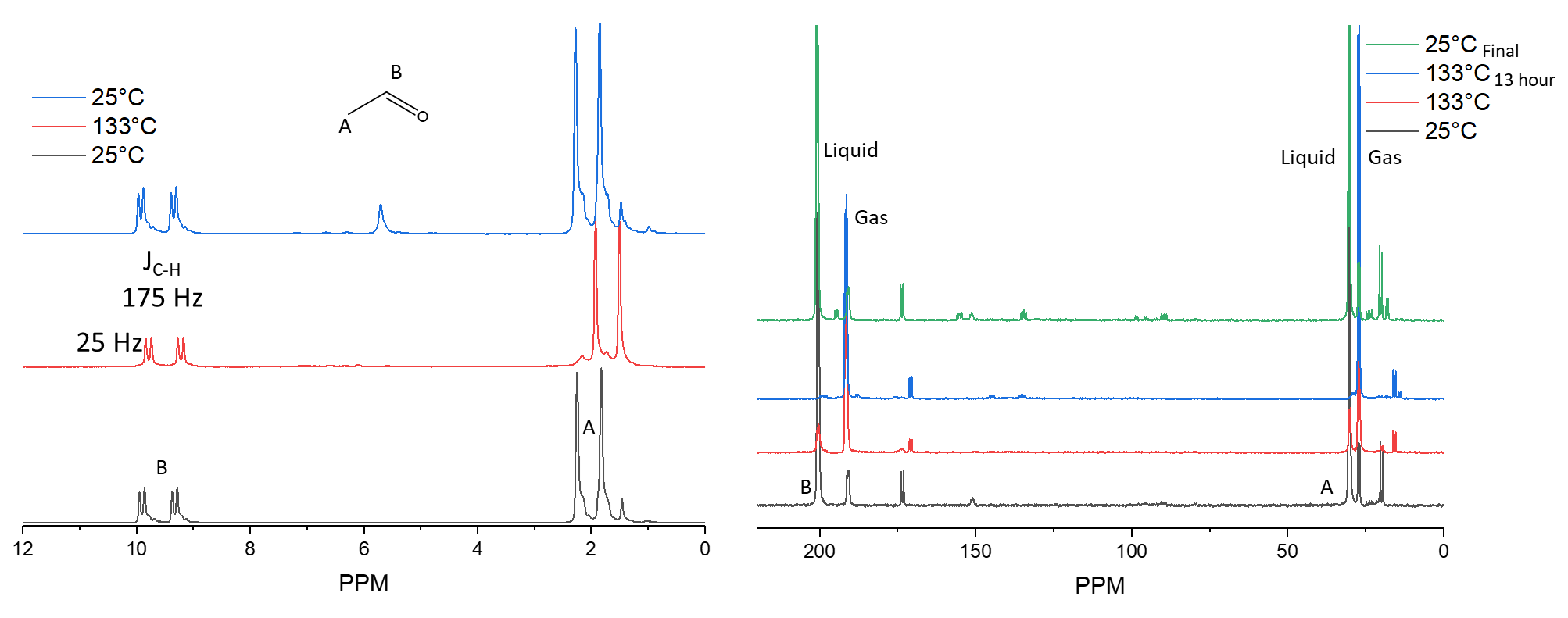


Figure S13. ^1^H (left) and ^13^C (right) MAS NMR spectra of 20 μL ^13^C-acetaldehyde in N_2_.


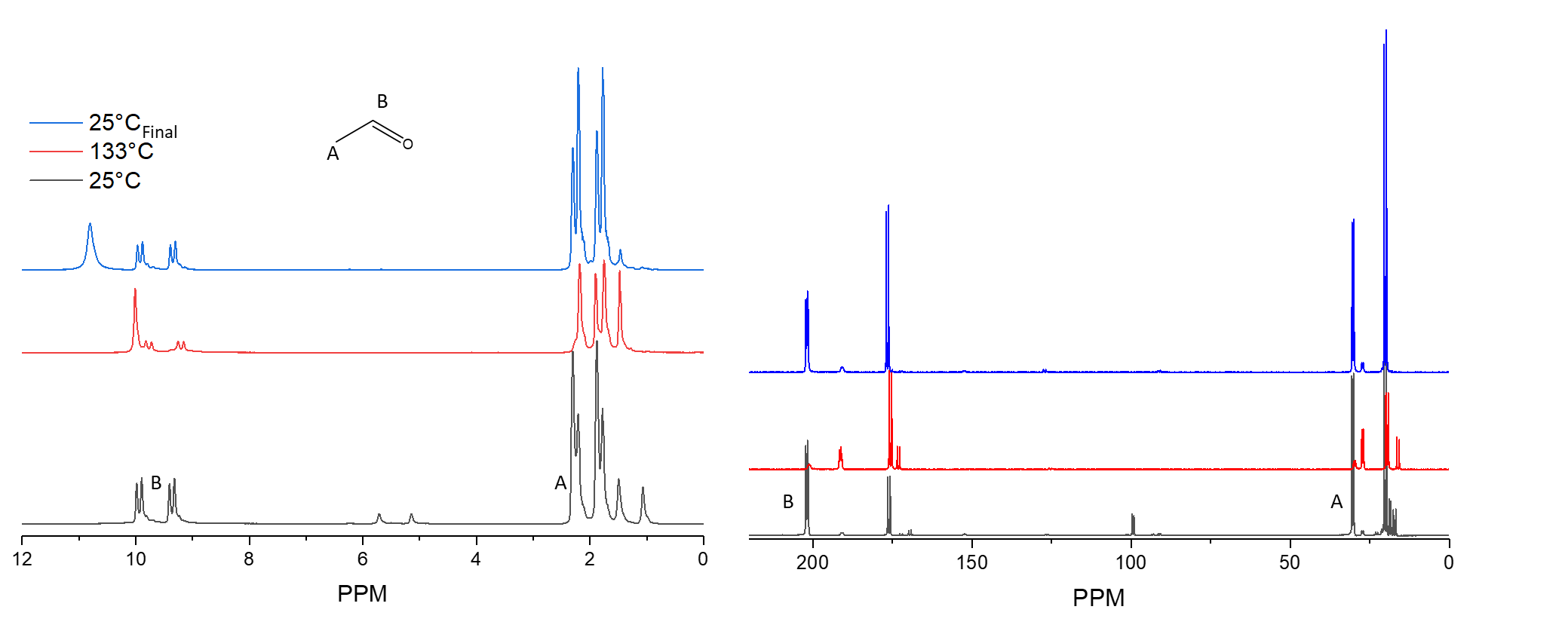


Figure S14. ^1^H (left) and ^13^C (right) MAS NMR spectra of 20 μL ^13^C-acetaldehyde in 76 psig O_2_.


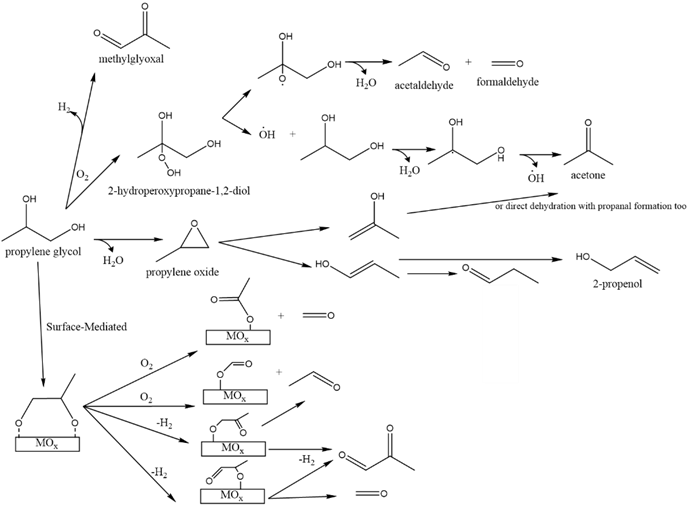


Figure S15. Proposed decomposition pathways for propylene glycol.

Figure S16. Pathway for propylene glycol radical formation.


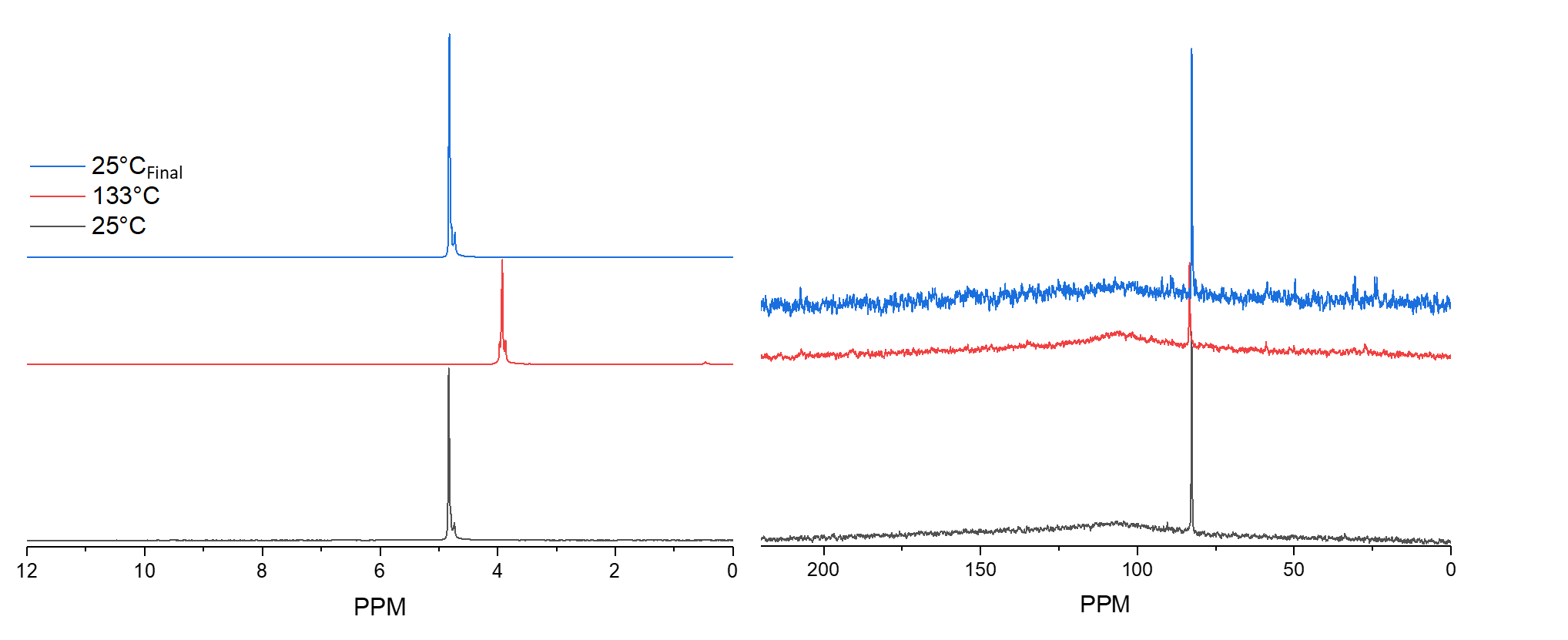


Figure S17. ^1^H (left) and ^13^C (right) MAS NMR spectra of 20 μL 0.5% acrolein in H_2_O and N_2_.


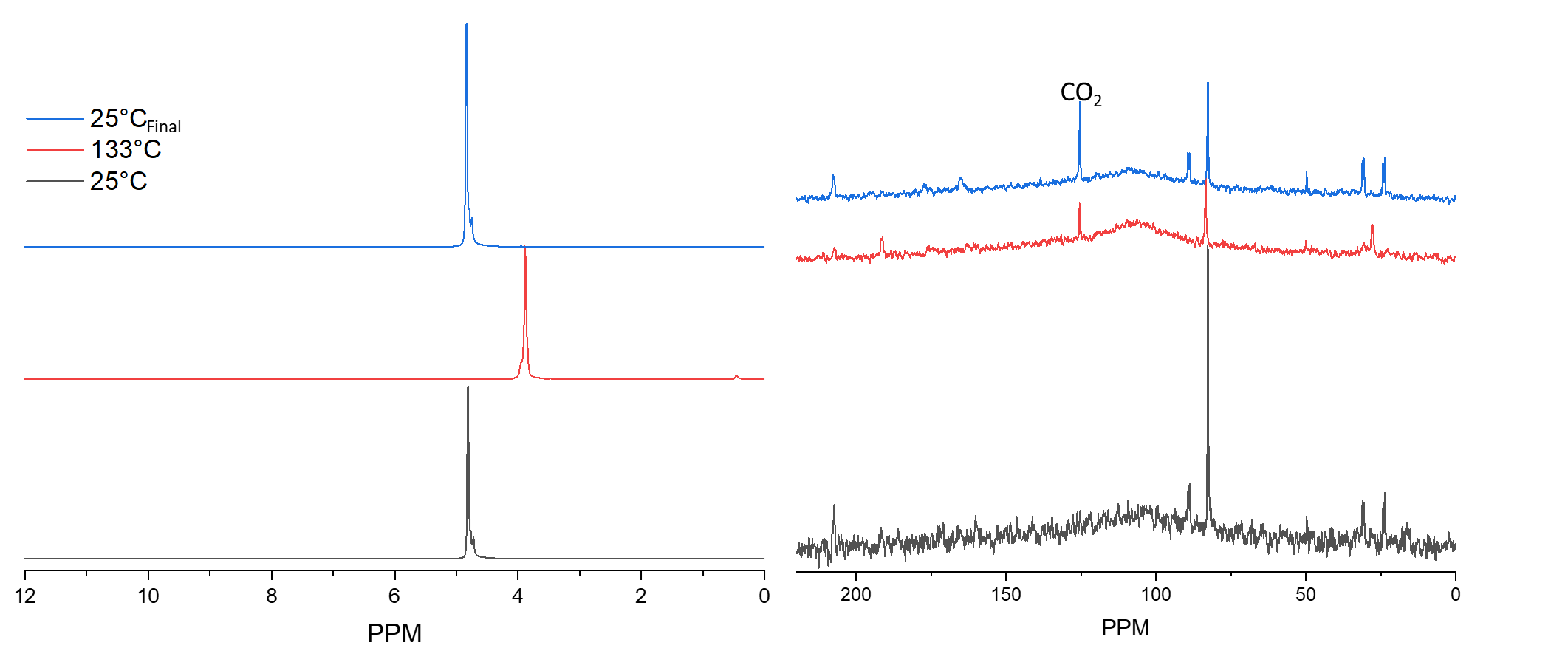


Figure S18. ^1^H (left) and ^13^C (right) MAS NMR spectra of 20 μL 0.5% acrolein in H_2_O and 75 psig O_2_. CO_2_ is the only peak in common with the mixed propylene glycol and glycerol experiment.


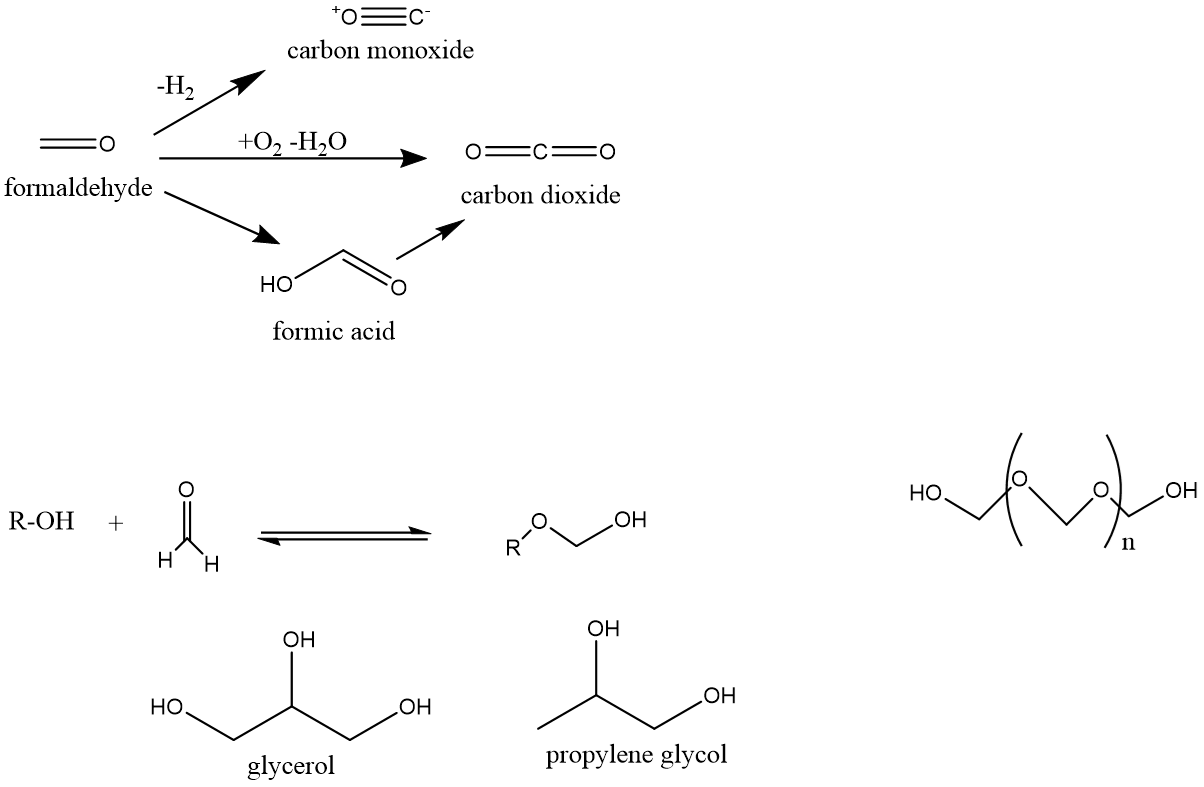


Figure S19. Decomposition pathway for formaldehyde.

Figure S20. Time sequence of in situ ^13^C MAS NMR of 20 μl glycerol (black), 20 μl propylene glycol (red), and a mixture 20 μl/20 μl (blue) in 75 psig O_2_.

Figure S21. ^13^C NMR spectrum of 18.7 mg Cr_2_O_3_ with 75 μL of propylene glycol, 75 μL of glycerol in the absence of O_2_ after thermal treatment at 175°C for 30 hours and 220°C for 10 hours.


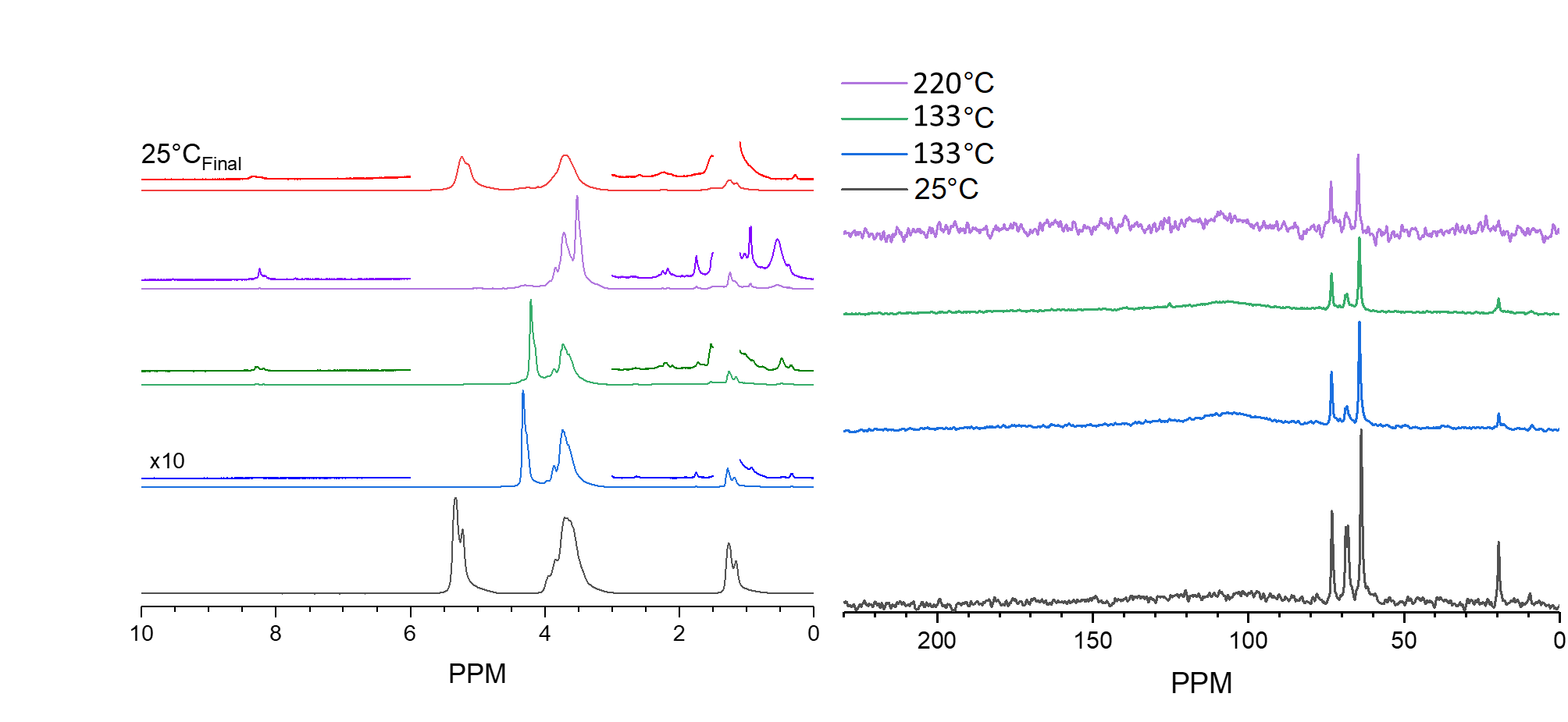


Figure S22. ^1^H (left) and ^13^C (right) MAS NMR spectra 86 mg Cr_2_O_3_ with 20 μL propylene glycol, 20 ul glycerol, and 75 psig O_2_.


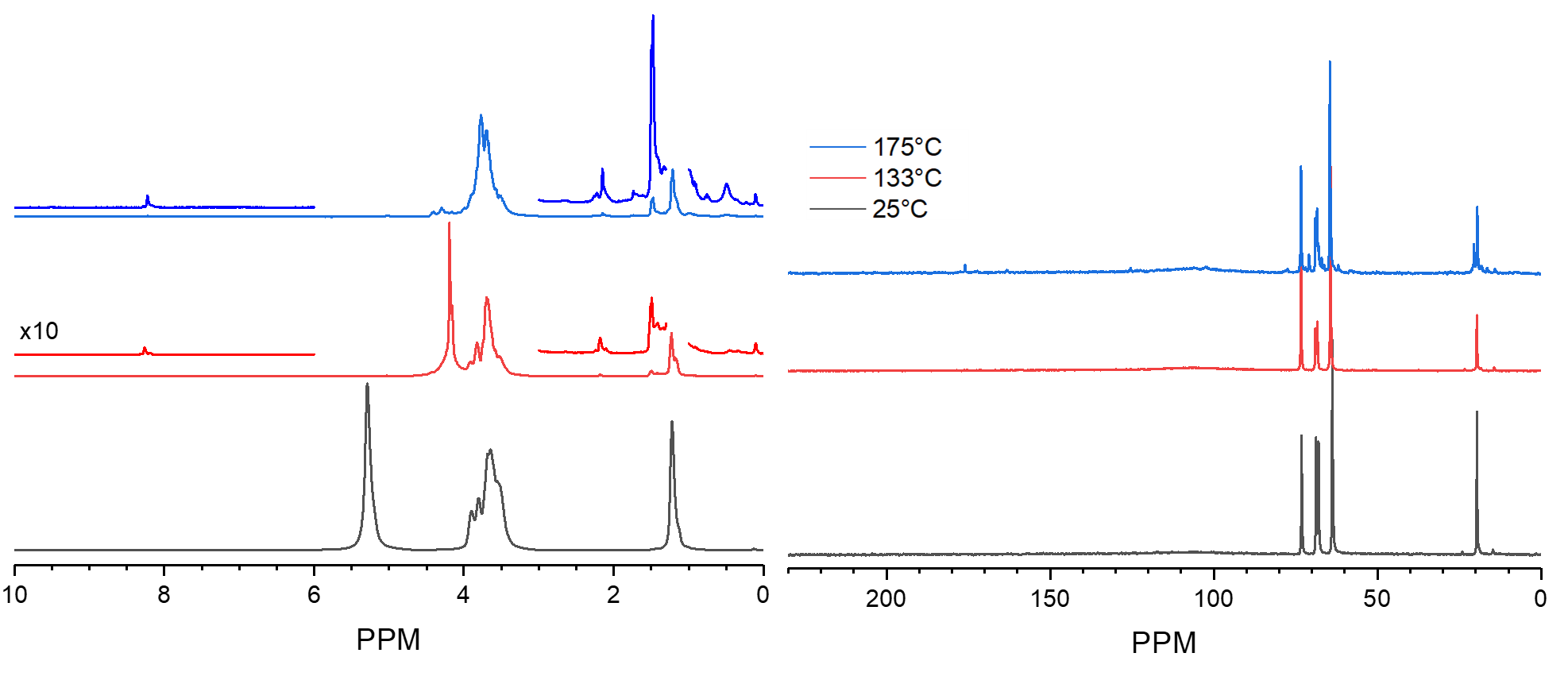


Figure S23. ^1^H (left) and ^13^C (right) MAS NMR spectra of 51.3 mg ZrO_2_ with 20 μL propylene glycol, 20 μl glycerol and 76 psig O_2_.
